# Epithelial SLC25A20 Deficiency Drives Immunosuppressive Neutrophil Remodeling via ARG1 Palmitoylation to Promote Colorectal Cancer

**DOI:** 10.1002/advs.78114

**Published:** 2026-10-04

**Authors:** Yue Han, Tian‐Yuan Tan, Bin Zhang, Ye‐Bin Fu, Wen Chen, Min‐Hui Yang, Yi‐Qing Wang, Yan‐Qing Ding, Shuang Wang

**Affiliations:** ^1^ Department of Pathology, Nanfang Hospital, School of Basic Medical Sciences Southern Medical University Guangzhou China; ^2^ Department of Pathology, Zhujiang Hospital Southern Medical University Guangzhou China; ^3^ Guangdong Province Key Laboratory of Molecular Tumor Pathology Guangzhou Guangdong China

**Keywords:** colorectal cancer, metabolic reprogramming, metastasis, palmitoylation, solute carrier family member 20, tumor‐associated neutrophils

## Abstract

While lipid metabolic reprogramming is increasingly recognized as a substantial driver of tumor microenvironment (TME) remodeling, how dysregulated fatty acid oxidation (FAO) contributes to immune evasion in colorectal cancer (CRC) progression remains obscure. Here, we identify the mitochondrial carnitine‐acylcarnitine translocase solute carrier 25 member 20 (SLC25A20), an essential conduit for FAO, as a key regulator of CRC progression through neutrophil phenotypic remodel. Mechanistically, SLC25A20 deficiency in CRC cells blunts mitochondrial long‐chain fatty acid (LCFA) import and FAO, triggering the release of LCFAs into the TME. This lipid‐rich niche not only vigorously recruits tumor‐associated neutrophils (TANs), but intrinsically programs them toward an immunosuppressive phenotype. Specifically, tumor‐derived LCFAs enforce arginase‐1 (ARG1) protein stability in TANs via ZDHHC18‐mediated palmitoylation. This distinct post‐translational modification (PTM) is indispensable for TAN‐mediated CD8^+^ T cell suppression and subsequent tumor immune evasion. Importantly, genetic or pharmacological blockade of this palmitoylation axis in TANs reinvigorates antitumor immunity and abolishes the tumor‐promoting effects of SLC25A20 deficiency. Collectively, our findings uncover a novel lipid‐driven metabolic symbiosis between CRC and neutrophils, highlighting the ZDHHC18‐ARG1 palmitoylation axis as a promising therapeutic strategy for CRC intervention.

## Graphical abstract

SLC25A20 deficiency in tumor cells compromises mitochondrial LCFA transport, which suppresses FAO and drives compensatory release of palmitic acid‐enriched lipids into the TME. These tumor‐derived palmitic acids are taken up by TANs, promoting ZDHHC18‐catalyzed ARG1 palmitoylation. Palmitoylation‐driven ARG1 accumulation in TANs substantially inhibits CD8^+^ T‐cell proliferation, impairs antitumor immunity, and consequently accelerates CRC progression.

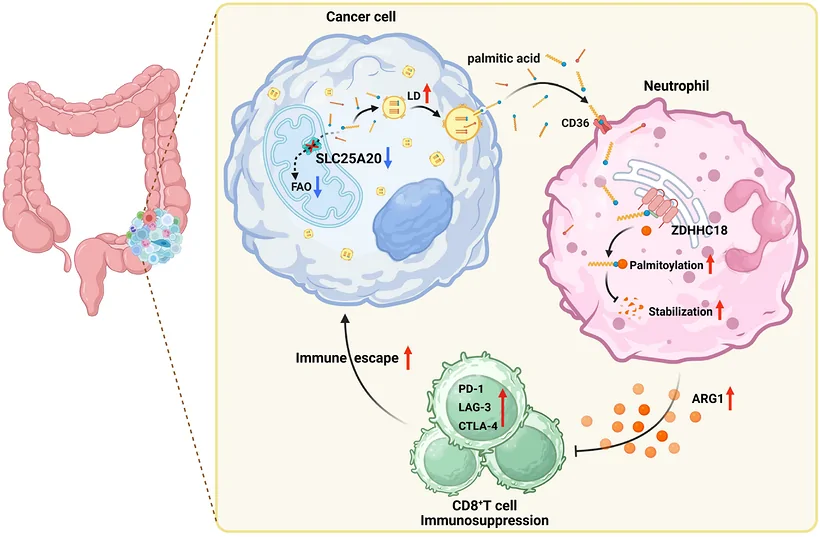

## 1 Introduction

Colorectal cancer (CRC) is the third most common malignancy and the second leading cause of cancer‐related death worldwide [1]. Epidemiological evidence suggests that modifiable lifestyle choices, such as a high‐fat diet, tobacco use, obesity, and a lack of exercise, significantly increase the incidence of CRC [2]. Despite therapeutic advances, such as surgery, chemotherapy, targeted agents, and immune checkpoint inhibitors, CRC mortality remains substantial, mainly driven by distant metastasis or disease recurrence [3]. Therefore, a deeper understanding of the molecular mechanisms driving malignant progression, along with the development of novel therapeutic strategies for CRC, is urgently needed.

Accumulating evidence demonstrates that immune cells in the tumor microenvironment (TME) play crucial roles in cancer progression and clinical outcomes [4, 5, 6]. Among immune components, tumor‐associated neutrophils (TANs) have emerged as key contributors to cancer evolution [7, 8, 9]. While TANs were traditionally classified into antitumor (N1) and protumor (N2) states [10], similar to tumor‐associated macrophages (TAMs), this dichotomy is now recognized as an oversimplification of their diversity in the TME [11]. Indeed, recent high‐resolution mapping in human CRC has defined a refined framework of four distinct TAN subsets: transitional precursor (TAN1), antigen‐presenting anti‐tumor (TAN2/TAN3), and pro‐tumor phenotype (TAN4), each characterized by unique molecular signatures and spatial distributions [12]. Given that high neutrophil accumulation is a hallmark of CRC associated with advanced stage and poor clinical outcomes [13, 14, 15], deciphering the intricate interactions between TANs and CRC cells is crucial for identifying novel therapeutic targets. However, the precise mechanisms governing TAN function in CRC remain to be fully elucidated.

Metabolic reprogramming is a hallmark of malignancies [6], with cancer cells demonstrating metabolic plasticity to withstand and grow in a harsh TME [16]. Lipid metabolism is typically upregulated throughout cancer progression [17], serving not only to produce energy but also to trigger oncogenic signaling, epigenetic events, and changes in immune cells, thereby remodeling immunosuppressive environments that favor tumor growth and metastasis [18]. Fatty acid β‐oxidation (FAO) is a multistep mitochondrial catabolic process that breaks down long‐chain fatty acids (LCFAs) to produce acetyl‐CoA, ATP, and cytosolic NADPH [19]. A key regulatory hub for FAO is the carnitine‐acylcarnitine shuttle system, where FAs are converted to acylcarnitines by carnitine palmitoyltransferase I (CPT1), followed by the translocation of acylcarnitines into the mitochondrial matrix via SLC25A20 (a carnitine/acylcarnitine translocase, CACT), and reconversion into acy‐CoAs catalyzed by CPT2. Although enhanced FAO is a broad feature of cancer [20, 21], different tumor types exhibit unique metabolic adaptations that remodel of their specific lipidome [22, 23, 24, 25]. However, the precise role of FAO in various cancers, particularly how dysregulation contributes to CRC progression, is not fully understood.

Here, we identify SLC25A20, an essential translocase for FAO, as a key prognostic modulator that is significantly downregulated in both CRC tissues and cells. Notably, SLC25A20 deficiency in tumor cells promotes CRC progression by eliciting a neutrophil‐mediated immunosuppressive response. Mechanistically, we demonstrated that SLC25A20 depletion impairs mitochondrial LCFA transport, leading to attenuated FAO and a subsequent surge in extracellular release of palmitic acid‐enriched LCFAs into the TME. These palmitic acids are internalized by TANs, where they promote zinc finger DHHC‐type palmitoyltransferase 18 (ZDHHC18)‐mediated arginase 1 (ARG1) palmitoylation. This modification enhances ARG1 protein stability, ultimately forging an immunosuppressive TME that facilitates CRC progression. Furthermore, the inhibition of ARG1 palmitoylation in TANs or LCFA production in tumor cells effectively suppresses CRC growth. Collectively, our findings reveal a novel SLC25A20‐dependent mechanism wherein lipid metabolic rewiring in CRC cells drives polarization of immunosuppressive TANs, providing potential therapeutic vulnerabilities for CRC intervention.

## 2 Results

### 2.1 SLC25A20 is Significantly Downregulated in CRC and Its Deficiency Predicts a Poor Prognosis

Given that lipid metabolism is dynamic and complex, we first evaluated the expression patterns of FAO‐related genes across multiple cancer types using publicly available datasets from The Cancer Genome Atlas (TCGA). Intriguingly, compared with normal samples, most FAO‐related genes were significantly downregulated across various tumors (Figure 1A). Notably, SLC25A20, which encodes a carrier protein essential for the mitochondrial influx of acylcarnitines during FAO, was consistently downregulated in 17 malignancies, with the most substantial suppression observed in CRC (Figure 1A). Furthermore, Gene Set Enrichment Analysis (GSEA) of the TCGA cohort revealed that SLC25A20 downregulation was closely linked to tumor invasion and metastasis (Figure S1A and B). The previous studies have sporadically reported SLC25A20 is downregulated in prostate cancer, bladder cancer, and hepatocellular carcinoma (HCC) [23, 26, 27]. In addition, the SLC25A20 rs7623023 polymorphism has been confirmed to be associated with an increased risk of CRC incidence associated with the consumption of red and processed meat [28]. Nevertheless, its specific role in CRC remains poorly understood. Prompted by these findings, we sought to focus on SLC25A20 and explore its specific role in CRC progression.

**FIGURE 1:**
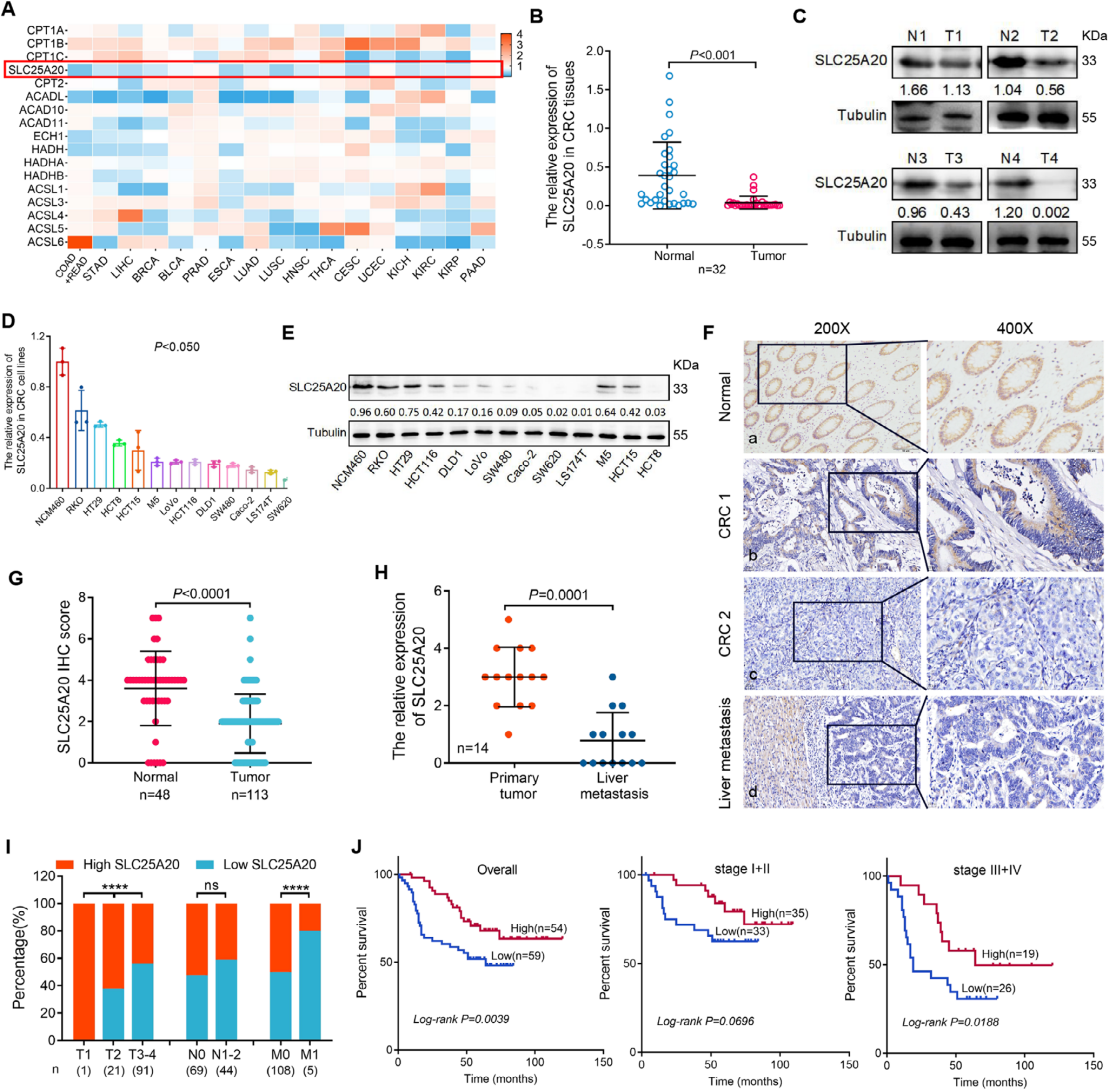
SLC25A20 is downregulated in CRC, especially in metastatic CRC, and associated with a positive prognosis for patients. (A) Hierarchical clustering analysis of FAO‐related gene expression in 17 malignances. (B and C) *SLC25A20* mRNA (B, n = 32) and protein quantification (C, n = 24) in CRC and its paired adjacent noncancerous tissues. (D and E) *SLC25A20* mRNA (D, n = 3) and protein (E) levels in a colon mucosa epithelial cell line (NCM460) and CRC cell lines. An anti‐tubulin antibody was employed for normalization in C and E. (F) SLC25A20 protein expression levels in normal colorectal mucosa and CRC tissues by IHC. (a, High SLC25A20 expression in normal colorectal tissue; b and c, CRC tissue with different SLC25A20 expression levels; d, Low SLC25A20 expression in liver metastases, with liver tissue on the left). Scale bars: 50 µm (200×) and 20 µm (400×). (G) IHC score of SLC25A20 expression in normal colorectal mucosa and CRC tissue (normal, n = 48; tumor, n = 113). (H) IHC score of SLC25A20 expression in primary tumors and its paired liver metastases from G (n = 14). (I) The correlation between SLC25A20 protein abundance and tumor TNM staging. (J) Kaplan‐Meier curves illustrating overall‐survival data for CRC patients (high expression, n = 54; low expression, n = 59) stratified according to SLC25A20 expression levels. Survival analyses are presented for the total patient cohort, the stage I‐II subgroup, and the stage III‐IV subgroup. Original uncropped and unaltered blots for this panel are provided in Figure S14. Data are represented as mean ± SD. Statistical significance was calculated by unpaired two‐sided t test (D and G), paired two‐sided t test (B and H) and log‐rank test (J); the *P* values are shown.

We next assessed the endogenous expression levels of SLC25A20 in CRC tissues and cell lines. As expected, both mRNA and protein levels of SLC25A20 were significantly diminished in CRC tissues compared to the matched surrounding noncancerous tissues (*P*<0.001, Figure 1B and C and Figure S1C). Similarly, SLC25A20 expression was consistently decreased across a panel of 12 CRC cell lines relative to the normal colon mucosal epithelial cell line, NCM460 (Figure 1D and E). Consistently, immunohistochemistry (IHC) staining also demonstrated reduced SLC25A20 protein expression in primary CRC tissues, with a further reduction observed in CRC‐derived liver metastasis foci (Figure 1F–H). Notably, clinicopathological correlation analysis revealed that SLC25A20 depletion was associated with advanced tumor stages (Figure 1I) and poor survival outcomes for CRC patients (Figure 1J). Subgroup survival analysis revealed that elevated SLC25A20 expression significantly correlated with improved overall survival in patients with advanced CRC (stages III/IV), but not in those with early‐stage disease (stages I/II, Figure 1J). These results suggest that SLC25A20 depletion is closely associated with CRC malignant progression and unfavorable prognosis in patients with advanced CRC.

### 2.2 SLC25A20 Deficiency in Tumor Cells Promotes CRC Progression via Neutrophil‐Mediated Immune Response

To investigate the biological functions of SLC25A20 in CRC, we established stable SLC25A20‐overexpressing cell lines using lentiviral‐based transfection in SW620 and LS174T cells, which display low endogenous expression. Conversely, we employed the CRISPR‐Cas9 system to knock out (KO) SLC25A20 in human RKO and HCT116 cells, which exhibit relatively high endogenous expression, as well as in mouse MC38 cells. Successful modulation of SLC25A20 expression was validated at both the mRNA and protein levels (Figure S2A–D). Next, SLC25A20‐KO cells and their wild‐type (WT) counterparts were subcutaneously injected into murine models. Notably, while there was no discernible difference in tumor growth between SLC25A20‐KO and control tumors in nude mice (Figure 2A and Figure S3A and B), SLC25A20 deletion in MC38 cells resulted in significantly accelerated tumor growth and increased tumor burden in C57BL/6 mice compared to the control (Figure 2B and Figure S3C and D). In line with these findings, SLC25A20‐KO markedly enhanced lung and liver metastasis in a C57BL/6 mouse model, whereas this effect was abrogated in immunodeficient nude mice (Figure 2C–E). Overall, these divergent outcomes between the two models suggest that the protumorigenic effect of SLC25A20 deficiency is dependent on alterations within the TME, particularly the tumor immune microenvironment (TIME).

**FIGURE 2:**
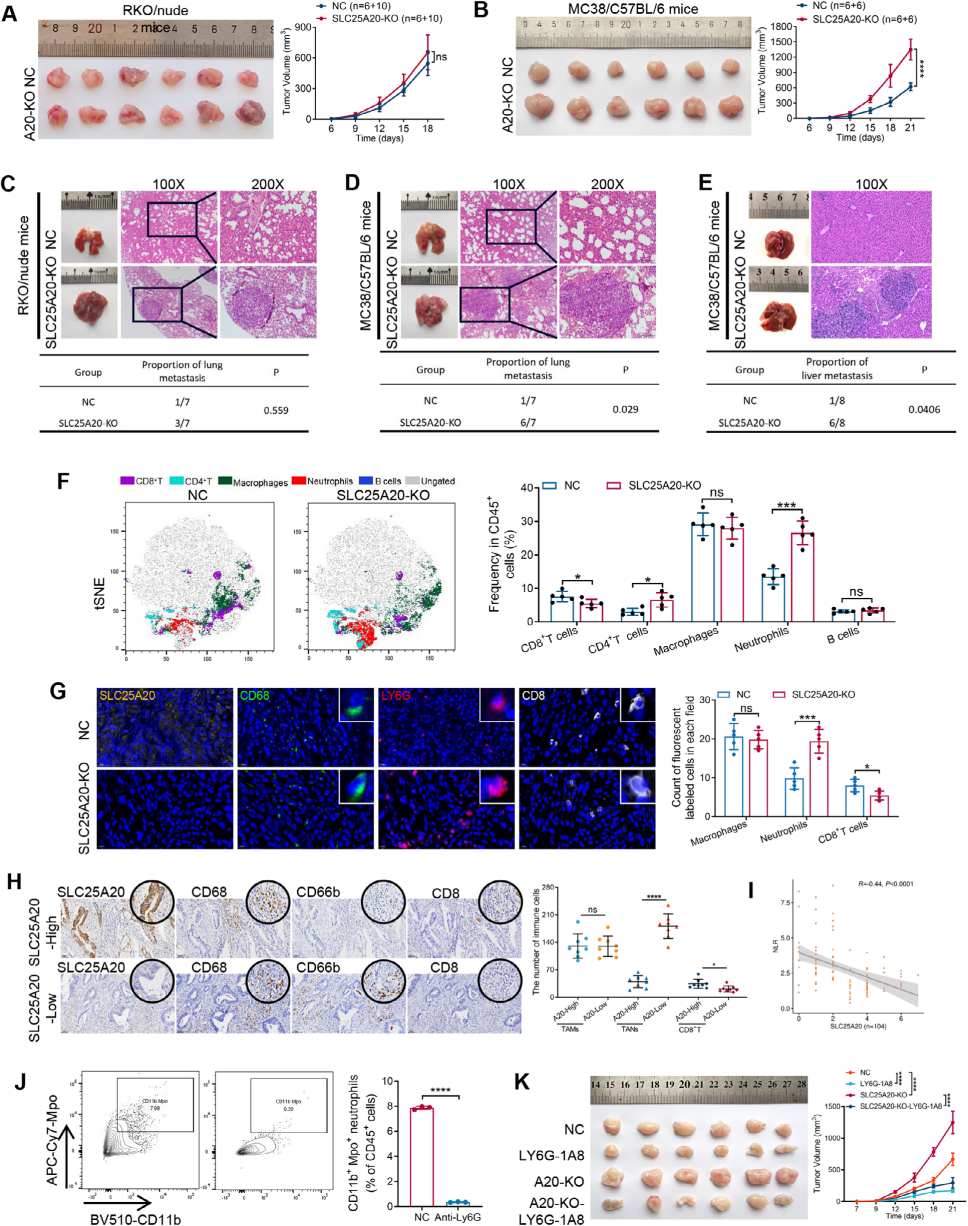
SLC25A20 restrains the tumorigenicity and metastasis of CRC cells in vivo and exerts more substantial effects in immunocompetent mice than in nude mice. (A) Representative images of subcutaneous tumors (left) and growth curves of tumor volume (right) from nude mice bearing subcutaneous xenografts of SLC25A20 knockout or control RKO cells (n = 6 + 10) for 18 days. (B) Representative images of subcutaneous tumors (left) and growth curves of tumor volume (right) from C57BL/6 mice, bearing subcutaneous xenografts of SLC25A20 knockout or control MC38 cells (n = 6 + 6) for 21 days. (C) Bright‐field images of lung metastases (upper left), representative images of H&E staining (upper right), and statistical comparisons (lower) from nude mice with lung metastases after tail vein injection of SLC25A20 knockout or control RKO cells (n = 7 per group). (D) Bright‐field images of lung metastases (upper left), representative images of H&E staining (upper right), and statistical comparisons (lower) from C57BL/6 mice with lung metastases after tail vein injection of SLC25A20 knockout or control MC38 cells (n = 7 per group). (E) Bright‐field images of liver metastases (upper left), representative images of H&E staining (upper right), and statistical comparisons (lower) from C57BL/6 mice with hepatic metastases after intrasplenic injection of SLC25A20‐knockout or control MC38 cells (n = 8 per group). (F) t‐distributed stochastic neighbor embedding (t‐SNE) visualization of tumor‐infiltrating immune cells isolated from mouse subcutaneous tumors. Five immune cell clusters were defined based on signature marker expression; clusters are color‐coded as indicated. (G) Representative fluorescence imaging staining of SLC25A20 (yellow), CD68 (green), LY6G (red), CD8 (white) and nuclear (DAPI, blue) (left) and statistical analysis (right, n = 5) in subcutaneous tumor tissues of C57BL/6 mice. (H) IHC detection of major immune‑cell populations in human CRC tissues with different SLC25A20 expression levels (CD68 labeling macrophages, CD66b labeling neutrophils, CD8 labeling CD8^+^ T cells). Statistical comparisons of macrophages, neutrophils and CD8^+^ T cells displayed on the right. (I) The correlation between SLC25A20 expression and NLR in CRC patients. (J) Flow‐cytometric quantification of tumor‐infiltrating neutrophils using MPO as a marker to verify neutrophil‐depletion efficiency in mice subjected to neutrophil‐depleting antibody treatment. Representative flow‐cytometry plots and corresponding statistical analysis are shown (n = 3 per group). (K) Representative images of subcutaneous tumors (left) and growth curves of tumor volume (right) from C57BL/6 mice subcutaneously injected with SLC25A20 knockout or control MC38 cells (n = 6 per group), and LY6G‐1A8 antibody was administered concomitantly to deplete neutrophils. Quantification displayed on the right. Scale bars, 100 µm or 50 µm. ns denotes nonsignificant. Data are presented as the mean ± SD. Statistical significance was calculated by two‐way ANOVA (A, B and K), unpaired two‐sided t test (F, G, H and J), Pearson correlation (I) and Fisher's exact test (C‐E). *P < 0.05, **P < 0.01, ***P < 0.001, and ****P < 0.0001.

To further delineate the specific immune cell alterations driven by SLC25A20 expression, we profiled the immune cell composition of MC38‐derived tumors from C57BL/6 mice using multicolor flow cytometry. Our analysis revealed a substantial expansion of CD11b^+^Ly6G^+^ neutrophils and a concurrent slight reduction in CD8^+^ T cells within SLC25A20‐KO tumors, while the abundance of CD11b^+^F4/80^+^ macrophages remained unchanged (Figure 2F). These immune phenotypic shifts were further corroborated by multiplex immunofluorescence (mIF) analysis of subcutaneous tumor tissues (Figure 2G). Parallel observations were made in clinical samples. Human CRC tissues with low SLC25A20 expression exhibited significantly enhanced TAN infiltration alongside a marginal decrease in CD8^+^ T cells (Figure 2H). Intriguingly, the expression level of SLC25A20 negatively correlated with peripheral neutrophil‐to‐lymphocyte ratio (NLR) from CRC patients, a biomarker that reflects the interaction between the immune system and disease processes and predicts cancer prognosis [29, 30], as shown in Figure 2I. To determine whether TANs are responsible for the protumorigenic effects associated with SLC25A20 deficiency in CRC, we pharmacologically depleted neutrophils in tumor‐bearing C57BL/6 mice using an anti‐Ly6G neutralizing antibody (1A8). IHC staining and flow cytometry targeting MPO were performed to confirm neutrophil depletion efficiency (Figure 2J and Figure S3E). Remarkably, neutrophil depletion attenuated the accelerated tumor progression induced by SLC25A20‐KO (Figure 2K). Collectively, these data demonstrate that TAN‐dominated remodeling of the TIME is indispensable for CRC progression mediated by SLC25A20 downregulation.

### 2.3 Tumoral SLC25A20 Deficiency Drives TAN Recruitment via FAO Impairment‐Induced Lipid‐Rich Microenvironments in CRC

Given that SLC25A20 functions as a mitochondrial carnitine‐acylcarnitine translocase essential for FAO, we hypothesized that its depletion promotes TAN infiltration by triggering intrinsic lipid metabolic rewiring within CRC cells. Initially, we observed that both the expression of key genes governing LCFA FAO and the overall FAO levels were significantly diminished in SLC25A20‐KO cells (Figure 3A and B). Subsequent lipidomic analysis further corroborated a intracellular accumulation of LCFAs (chain length > 12 carbons) following SLC25A20 ablation (Figure S4A), confirming a blockade of LCFA oxidation. Consistently, assessment of mitochondrial FAO capacity via palmitate‐dependent oxygen consumption rate (OCR) revealed a impairment in SLC25A20‐KO cells. Notably, the magnitude of this OCR defect was comparable to that induced by etomoxir, a well‐established CPT1 inhibitor (Figure 3C).

**FIGURE 3:**
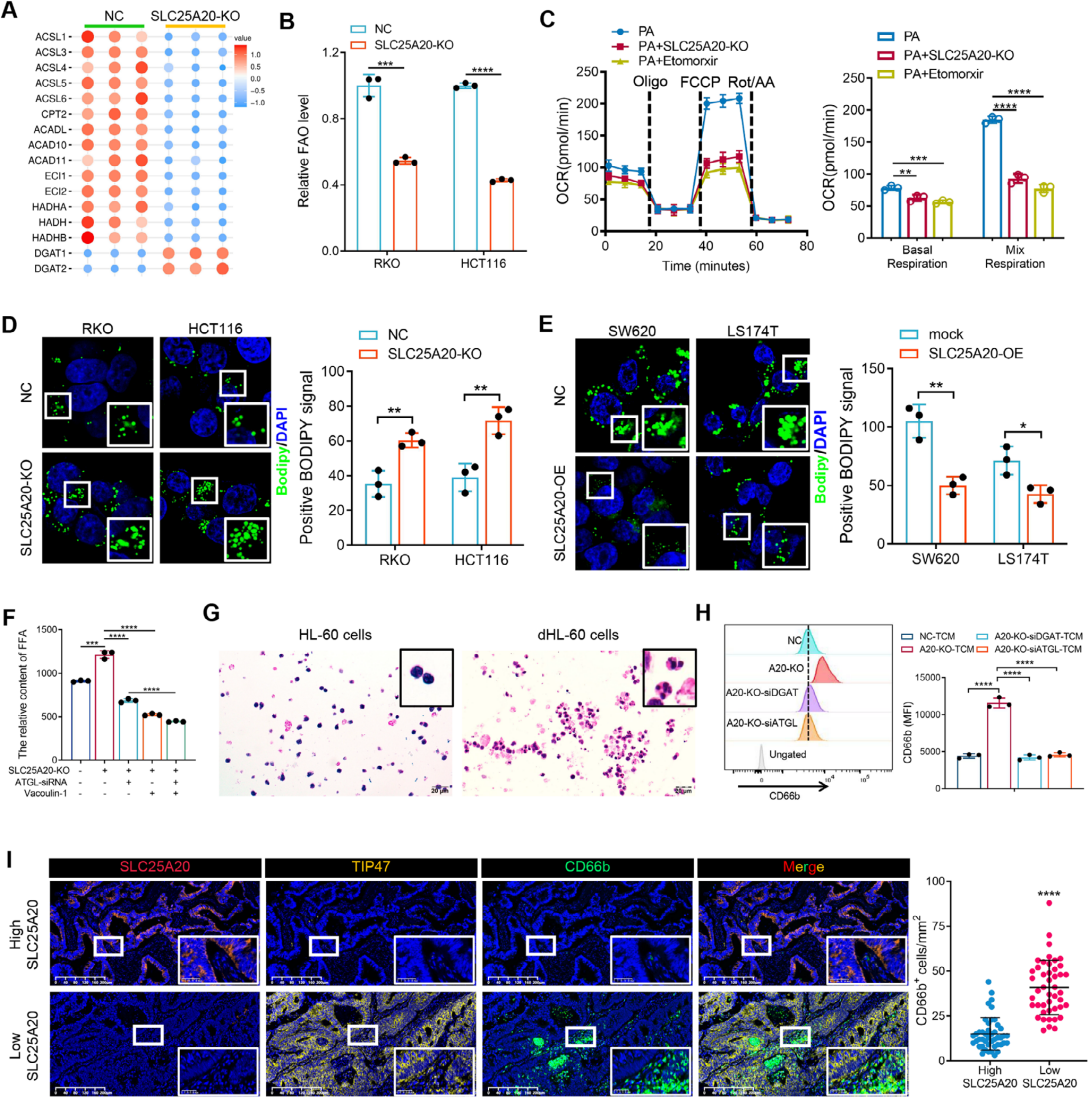
SLC25A20 deficiency in CRC cells stimulates lipid metabolism reprogramming manifested by releasing FAs to the TME. (A) Transcript levels of LCFA‐associated FAO and LD biogenesis‐related genes in control and SLC25A20 knockout RKO cells were quantified by real‐time PCR (n = 3 biologically independent experiments). (B) The FAO levels in SLC25A20 knockout and control RKO and HCT116 cells (n = 3). (C) OCR was determined under basal condition and additional administration of etomoxir by a Seahorse XF24 analyzer in control and SLC25A20 knockout RKO cells (left), n = 3. Analysis of basal respiration and max respiration (right). (D and E) Representative fluorescence imaging of LDs stained with BODIPY 493/503 (green) (left) and statistical analysis (right, n = 3) in SLC25A20 knockout (D), overexpression (E) and corresponding control cells. Nuclei were stained with DAPI (blue). (F) The FFA levels in SLC25A20 knockout and control RKO cells treated with ATGL‐siRNA or Vacuolin‐1 (n = 3). (G) Representative images of 0 (left) and 8 (right) days differentiated HL‐60 cells stained with Papanicolaou. (H) Migration of day‐8 differentiated dHL‐60 cells induced by culture supernatants from SLC25A20‐knockout RKO cells, with or without DGAT‐siRNA or ATGL‐siRNA transfection (n = 3). Relative CD66b abundance is presented as normalized MFI (ratio of stained to unstained controls) to correct for variable autofluorescence background. CD66b staining was used here to confirm the neutrophil‐like identity of migrated cells recovered from the lower Transwell chamber, as nearly all DMSO‐differentiated dHL‐60 cells exhibit CD66b positivity. (I) Representative fluorescence imaging staining of SLC25A20 (red), TIP47 (yellow), CD66b (green) and nuclear (DAPI, blue) in CRC tissues. The right panel presents the quantitative data and statistical comparisons. Data are presented as the mean ± SD. Statistical significance was calculated by unpaired two‐sided t test (B, D, E and I) and one‐way ANOVA with Tukey's post hoc test (C, F and H). *P < 0.05, **P < 0.01, ***P < 0.001, and ****P < 0.0001.

Generally, lipid droplet (LD) biogenesis serves as a adaptive response to impaired FAO [31]. In line with this, quantification of neutral lipid storage via BODIPY 493/503 staining demonstrated that SLC25A20‐KO robustly induced LD accumulation in CRC cells compared to the controls. Conversely, SLC25A20 overexpression curtailed the number of LDs (Figure 3D and E). This was accompanied by an increase in expression of diacylglycerol acyltransferase 1 (DGAT1) and DGAT2, two enzymes essential for triacylglycerol synthesis and LD formation (Figure 3A). The exacerbated LD accumulation in SLC25A20‐KO cells was further confirmed by Oil Red O staining (Figure S4B). To rule out increased extracellular lipid uptake as the primary driver, we evaluated CD36, a major membrane transporter responsible for the cellular influx of LCFAs [32]. SLC25A20‐KO did not modulate CD36 expression, and importantly, CD36 knockdown failed to alter the LD accumulation triggered by SLC25A20 depletion (Figure S4C–E). Collectively, these findings suggest that SLC25A20 deficiency impairs FAO, thereby redirecting intracellular lipids toward LD accumulation to maintain lipid homeostasis in CRC cells.

Intracellular LDs are recognized as dynamic organelles that regulate lipid storage, lipolysis, and release [31]. To determine whether SLC25A20 deficiency modulates fatty acid (FA) efflux following LD formation, we knocked down adipose triglyceride lipase (ATGL) or DGAT1/2 using siRNA to inhibit LD lipolysis or synthesis in CRC cells (Figure S4F). We observed a significant increase in FA levels in tumor‐conditioned media (TCM) from SLC25A20‐depleted CRC cells, an effect that was effectively abrogated by ATGL or DGAT1/2 silencing (Figure 3F and Figure S4G). Consistently, the treatment of Vacuolin‐1, an antagonist for lysosomal Ca^2+^ channel MCOLN1 which regulates the efflux of lipolysis‐derived FAs [33, 34], significantly inhibited the FA efflux induced by SLC25A20 deficiency. Importantly, the inhibitory effect of Vacuolin‐1 on FA efflux was further attenuated upon ATGL knockdown in CRC cells (Figure 3F). These findings support the conclusion that SLC25A20‐deficient CRC cells store impaired FAO‐derived FAs within LDs, which are subsequently released into the TME via a dynamic lipolytic and secretory program.

Tumor metabolic reprogramming is known to reshape the landscape and functionality of immune cells within the TME during malignant progression [35]. Given that lipids serve as potent regulators of neutrophil recruitment, function, and survival [36], we hypothesized that SLC25A20‐deficient CRC cells induce a lipid‐rich microenvironment to facilitate neutrophil infiltration. To test this hypothesis, we employed the human promyelocytic leukemia cell line (HL‐60 cells), a well‐validated in vitro model for investigating neutrophil biology [37]. Specifically, HL‐60 cells were differentiated into neutrophil‐like cells (dHL‐60) upon treatment with 1.25% dimethyl sulfoxide (DMSO) (Figure 3G and Figure S5A). Subsequent coculture assays demonstrated that TCM derived from SLC25A20‐deficient CRC cells significantly enhanced dHL‐60 cell migration, which could be effectively abrogated by inhibiting the synthesis and lipolysis of LDs (Figure 3H). Immunofluorescence staining of human CRC clinical specimens also revealed an inverse correlation of SLC25A20 expression with local lipid accumulation as well as TAN infiltration within the TME (Figure 3I). Collectively, these data suggest that SLC25A20 depletion in CRC cells drives the excessive release of FAs, which in turn contributes to TAN recruitment.

### 2.4 SLC25A20 Deficiency‐Induced FA Efflux Drives the Polarization of TAN Toward an Immunosuppressive Phenotype

To characterize the specific phenotypic landscape of TANs modulated by SLC25A20‐depleted CRC cells, we performed multilabel immunofluorescence staining on human CRC tissues. We observed a significant enrichment of CD66b^+^ ARG1^+^ TANs, indicative of an immunosuppressive phenotype, in regions with low SLC25A20 expression (Figure 4A). Furthermore, we conducted a lipid pulse‐chase assay to verify direct LCFA transfer from CRC cells to neutrophils. CRC cells were preincubated with a fluorescently labeled LCFA (BODIPY‐C_16_) and subsequently cocultured with dHL‐60 cells. Flow cytometry revealed a progressive increase in the BODIPY‐C_16_ signal within dHL‐60 cells, demonstrating the efficient transfer of lipids from tumor cells to neutrophils (Figure 4B and C). Next, to assess the functional consequences of this lipid transfer, dHL‐60 cells were exposed to TCM from SLC25A20‐deficient CRC cells in a coculture system (Figure 4D). This treatment induced a robust upregulation of immunosuppressive markers (ARG1, Ym‐1, and CD206) and a concomitant downregulation of the immunostimulatory marker IL‐1α. These phenotypic shifts were abrogated upon CD36 knockdown in dHL‐60 cells (Figure 4E and Figure S5B). Consistently, flow cytometry also revealed elevated normalized ARG1 MFI within CD66b‐positive dHL‐60 cells upon incubation with SLC25A20‐depleted TCM (Figure 4F). We also assessed the contribution of other tumor‐derived factors, including chemokines and TGF‐β to neutrophil reprogramming. No significant alterations in the levels of these factors were detected upon SLC25A20 deficiency (Figure S6). Collectively, these findings establish that FAs derived from SLC25A20‐depleted CRC cells serve as metabolic cues that are internalized by neutrophils, thereby driving their remodeling toward a protumorigenic, immunosuppressive phenotype.

**FIGURE 4:**
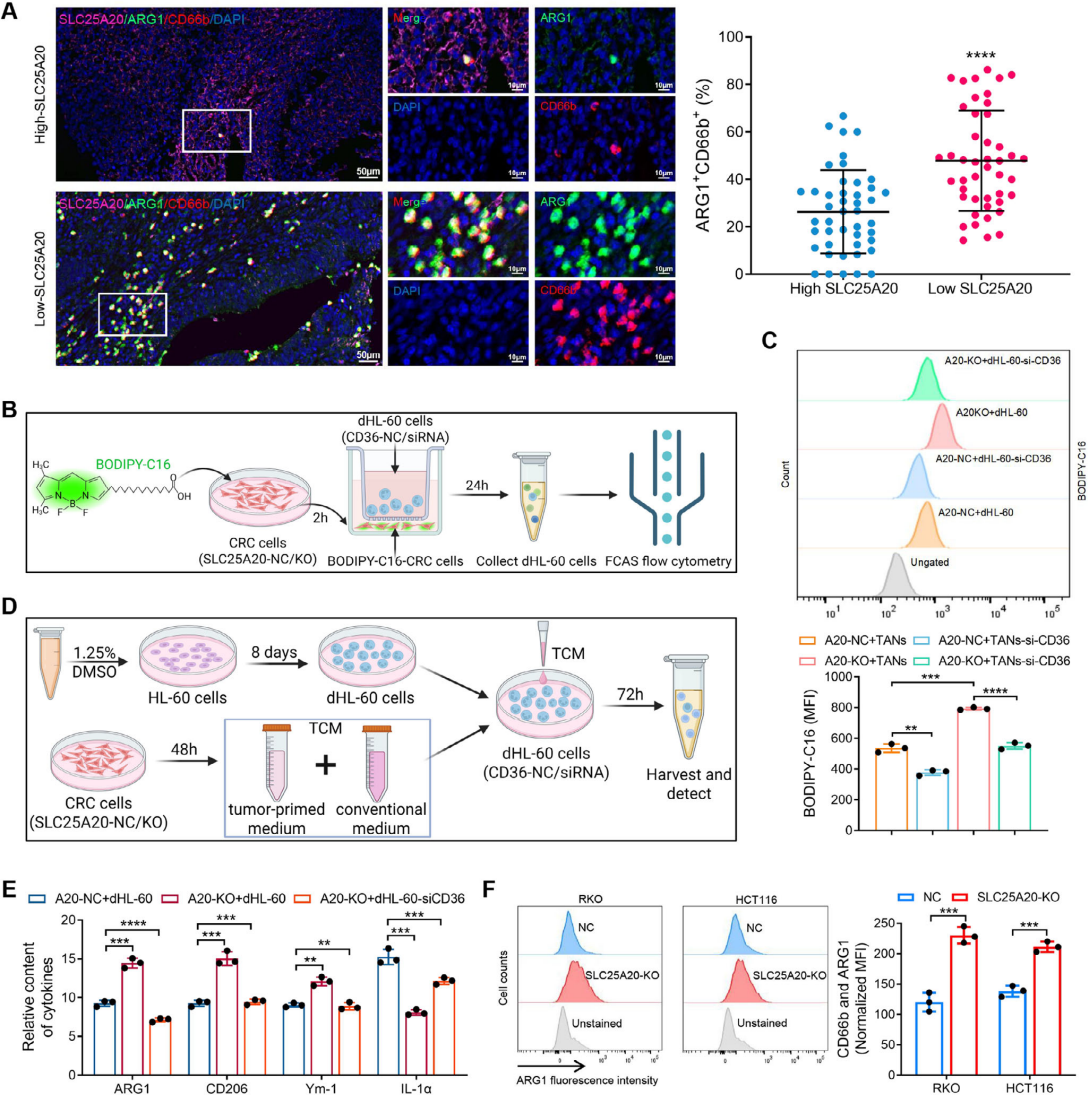
SLC25A20 downregulation promotes pro‐tumorigenic polarization of TANs by inducing lipid‐rich microenvironment formation. (A) Representative fluorescence imaging staining of SLC25A20 (purple), TIP47 (yellow), CD66b (red), ARG1 (green) and nuclear (DAPI, blue) in CRC tissues. The right panel presents the quantitative data and statistical comparisons. (B) Pattern diagram of co‐culture of BODIPY‐C16 labeled CRC cells with dHL‐60 cells. (C) dHL‐60 cells co‐cultured with BODIPY‐C16‐labelled CRC cells. Relative abundance of BODIPY‐C16 in dHL‐60 cells represented by normalized MFI (ratio of stained over unstained controls to correct for distinct autofluorescence background). (D) Pattern diagram of TCM acquisition and cultivation of dHL‐60 cells with it. (E) ELISA‐measured levels of immunosuppressive markers (ARG1, CD206, Ym‐1) and the immunostimulatory cytokine IL‐1α in day‐8 differentiated dHL‐60 cells. dHL‐60 cells were transfected with or without CD36‐siRNA and cultured with supernatants from SLC25A20‐knockout or control RKO cells (n = 3 biologically independent experiments). (F) Normalized MFI of ARG1 and CD66b within CD66b‐positive neutrophil‐like dHL‐60 cells was assessed in the co‐culture system of day‐8‐differentiated HL‐60 cells treated with supernatant from SLC25A20‐knockout or control RKO and HCT116 cells (n = 3). Representative flow‐cytometry histograms show cell counts (y‐axis) versus CD66b fluorescence intensity (x‐axis). Quantification panels display normalized mean fluorescence intensity (MFI) of CD66b and ARG1 measured in the CD66b‐positive neutrophil‐like population. Normalized MFI was calculated as the MFI of stained samples divided by the MFI of unstained negative controls to correct for cellular autofluorescence. Quantitative results displayed on the right. Values for n represent biologically independent samples. Data are presented as the mean ± SD. Statistical significance was calculated by one‐way ANOVA with Tukey's post hoc test (C and E) and unpaired two‐sided t test (A and F). **P < 0.01, ***P < 0.001, and ****P < 0.0001.

### 2.5 SLC25A20 Deficiency in Tumor Cells Enhances Palmitoylation‐Mediated ARG1 Protein Stability in TANs

Given that ARG impairs T cell responses by depleting L‐arginine and disrupting T cell receptor signaling [11], elevated ARG expression serves as a canonical hallmark of immunosuppressive TAN phenotype [10]. To elucidate the mechanistic basis by which SLC25A20 deficiency drives TAN remodeling toward the immunosuppressive phenotype, we analyzed dHL‐60 cells cultured with TCM derived from SLC25A20‐depleted CRC cells. Although moderate upregulation of ARG1 mRNA was observed, immunoblotting detected a much more pronounced increase in ARG1 protein abundance (Figure 5A and B), indicating that SLC25A20‐depleted CRC cells promoted ARG1 upregulation in TANs via post‐translational modification (PTM).

**FIGURE 5:**
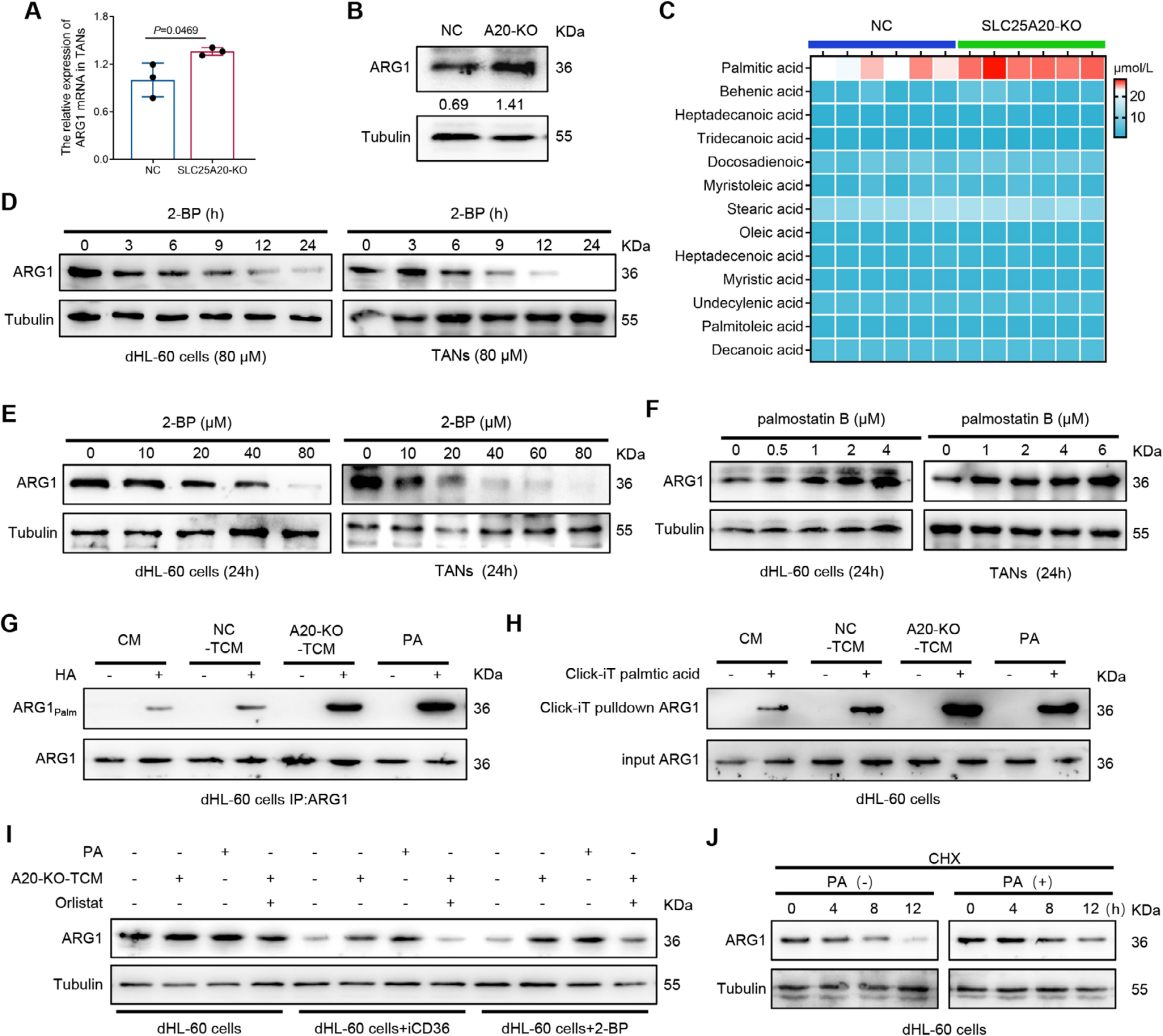
SLC25A20‐deficient CRC cells induce ARG1 palmitoylation in neutrophils. (A) The ARG1 transcript levels in dHL‐60 cells cultured with the supernatant of SLC25A20 knockout or control RKO cells were quantified by real‐time PCR (n = 3 biologically independent experiments). (B) Immunoblot analysis of ARG1 protein in dHL‐60 cells from A. Anti‐tubulin was used as a loading control. (C) Lipidomics analysis of differential lipid metabolites in supernatant from SLC25A20 knockout or control RKO cells (n = 6 per group). (D) dHL‐60 (left) cells and primary TANs (right) were cultivated with 80 µM 2‐BP for 1–24 h, followed by immunoblotting for ARG1. Anti‐tubulin served as a loading control. (E) dHL‐60 (left) cells and primary TANs (right) were treated with 2‐BP in different gradient concentration for 24 h, and ARG1 protein abundance was assessed by immunoblotting. (F) dHL‐60 (left) cells and primary TANs (right) were treated with Palmostatin B in different gradient concentration for 24 h; immunoblotting was performed to detect ARG1 and tubulin. (G) dHL‐60 cells were co‐cultured with supernatants from SLC25A20‐knockout or control RKO cells, with or without exogenous PA supplementation, for 72 h. ARG1 palmitoylation was detected by combining the ABE assay with immunoblot analysis. (H) dHL‐60 cells were co‐cultured with SLC25A20 knockout or control RKO cell supernatant or external PA supplementation for 72 h. ARG1 palmitoylation was assessed by click‐chemistry‐based labeling coupled with immunoblotting. (I) A co‐culture system of dHL‐60 cells (treated with CD36‐siRNA, 2‐BP or PA) and CRC cells (with/without SLC25A20 knockout, with/without Orlistat application) has been established. The levels of ARG1 were detected by western blots. An anti‐tubulin antibody was used for normalization. (J) dHL‐60 cells with or without palmitic acid administration were incubated with cycloheximide (CHX) and collected protein samples at designated time points, then immunoblot analysis of ARG1 protein by western blot was performed. The immunoblots in B and D‐J are representative of three independent experiments. Original uncropped and unaltered blots for this panel are provided in Figure S15. Data are presented as the mean ± SD. Statistical significance was calculated by unpaired two‐sided t test (A); the *P* values are shown.

As a pivotal mechanism for regulating protein function, PTMs orchestrate a myriad of physiological and pathological processes [38]. Given that metabolites exert noncanonical roles by mediating PTMs [39], we performed untargeted metabolomics to determine whether SLC25A20‐depleted CRC cells modulate neutrophil phenotype via specific metabolites and identified palmitic acid (C16:0) as the most significantly enriched free FA in TCM derived from SLC25A20‐depleted cells (Figure 5C and Figure S7A). Moreover, we found that exogenous palmitic acid regulated ARG1 expression in dHL‐60 cells in a dose‐dependent manner, which indicated that tumor‐derived palmitic acid may act as a key metabolic regulator of ARG1 in dHL‐60 cells (Figure S7B). Since palmitic acid serves as a substrate for S‐palmitoylation, a reversible lipid modification that often enhances protein stability via thioesterification of cysteine residues [40], we hypothesized that ARG1 might be a target of this modification. In dHL‐60 cells, pharmacological treatment with 2‐bromopalmitate (2‐BP), a broad‐spectrum inhibitor of DHHC‐mediated protein palmitoylation, led to a dose‐ and time‐dependent reduction in ARG1 protein levels (Figure 5D and E), whereas Palmostatin B, a selective inhibitor of APT‐dependent protein depalmitoylation, conversely increased ARG1 abundance (Figure 5F). Furthermore, primary TANs were isolated from WT and SLC25A20‐KO mouse tumor tissues by flow‐cytometric sorting and treated with 2‐BP or Palmostatin B. Consistent with our findings in dHL‐60 cells, TANs from SLC25A20‐KO tumors exhibited elevated ARG1 expression, which was similarly modulated by pharmacological targeting of palmitoylation (Figure 5D–F). In addition, acyl‐biotin exchange (ABE) assays were performed to detect palmitoylation in treated dHL‐60 cells. As expected, both SLC25A20‐depleted TCM coculture and exogenous palmitic acid significantly elevated the level of palmitoylated ARG1 in dHL‐60 cells (Figure 5G). Consistent with the ABE results, click‐chemistry assays confirmed the palmitoylation modification of ARG1 was increased by SLC25A20‐depleted TCM coculture (Figure 5H). Furthermore, the induction of ARG1 by SLC25A20‐depleted TCM was attenuated when the fatty acid synthase (FASN) in CRC cells was blocked by FASN inhibitor Orlistat, or when lipid uptake and palmitoylation in neutrophils were disrupted via CD36 knockdown or 2‐BP treatment (Figure 5I). Finally, a cycloheximide (CHX) chase assays revealed that 2‐BP accelerated the degradation of endogenous ARG1, while exogenous palmitic acid prolonged its half‐life (Figure 5J and Figure S7C). Taken together, these findings establish that SLC25A20 deficiency triggers the efflux of tumor cell‐derived palmitic acid, which is subsequently internalized by neutrophils to stabilize the ARG1 protein via a palmitoylation‐mediated pathway, thereby driving immunosuppressive TAN remodeling.

### 2.6 ZDHHC18 Specifically Catalyzes ARG1 Palmitoylation at Cys303 to Increase Its Stability via the Lysosomal Pathway in Neutrophils

To elucidate the basis underlying palmitoylation‐mediated ARG1 stabilization, we utilized the CSS‐Palm 4.0 algorithm (http://csspalm.biocuckoo.org) and identified a single putative palmitoylation site at cysteine 303 (Cys303) in ARG1 (Figure S8A and B). To validate this prediction, we generated an ARG1‐C303A mutant (Figure S8C and D). ABE assays showed that the C303A mutation completely abolished palmitic acid‐induced ARG1 palmitoylation (Figure 6A and B). To exclude endogenous ARG1 interference, we overexpressed FLAG‐tagged WT or C303A ARG1 in dHL‐60 cells, followed by anti‐FLAG immunoprecipitation and click‐chemistry‐based S‐acylation detection. The C303A mutant exhibited a reduced palmitoylation signal compared to the WT, confirming Cys303 as the primary palmitoylation site of ARG1 (Figure 6C). Furthermore, CHX chase assays revealed that the C303A mutant underwent accelerated degradation, mimicking the effect of 2‐BP treatment (Figure 6D). These results indicate that Cys303 is the predominant palmitoylation site of ARG1 and is indispensable for maintaining its protein stability.

**FIGURE 6:**
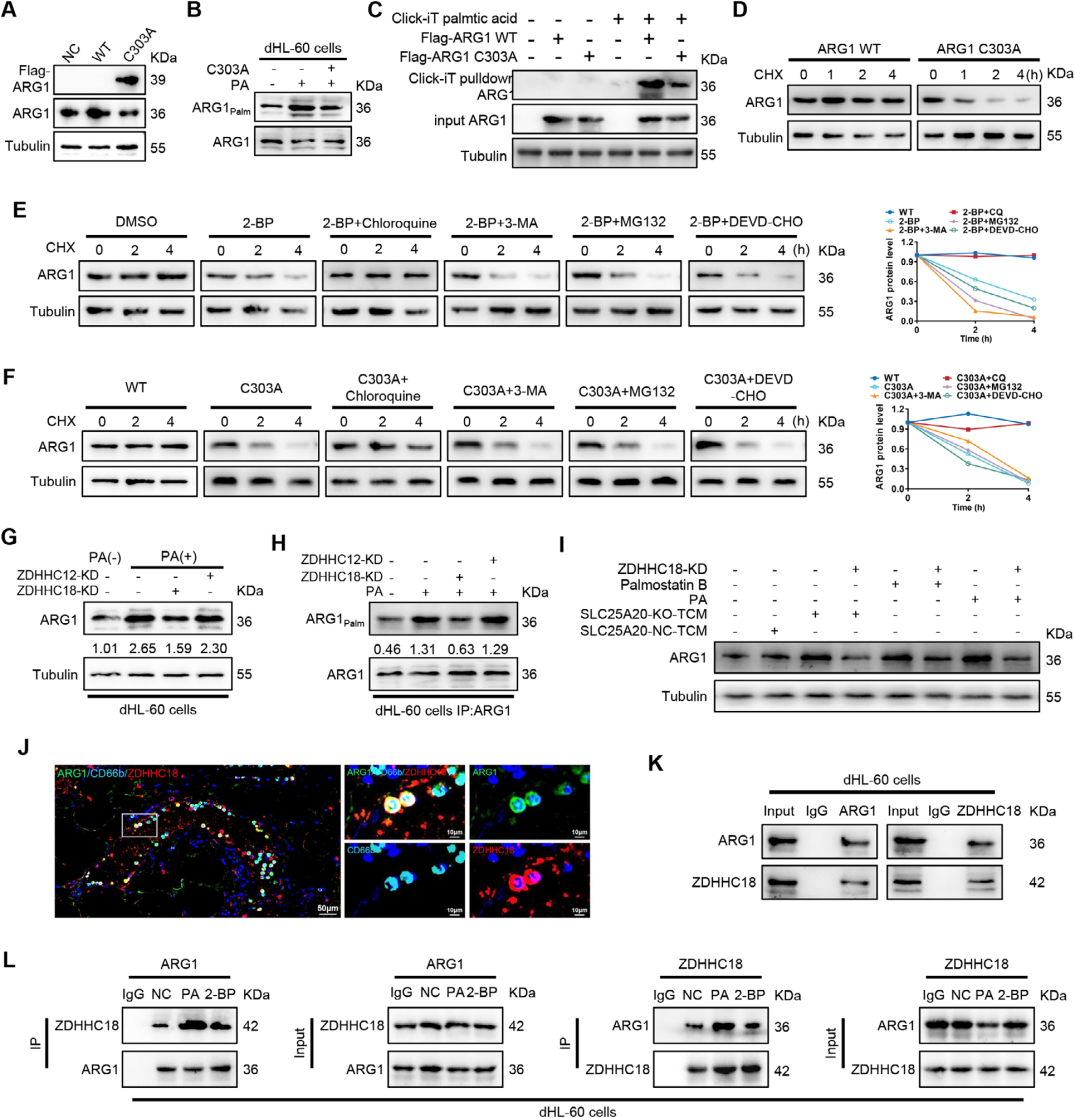
Identification of the palmitoylation site and palmitoyltransferase in ARG1. (A) dHL‐60 cells were transfected with wild‐type (WT) Flag‐ARG1 or the palmitoylation‐deficient Flag‐ARG1‐C303A mutant for 48 h. ARG1 protein expression were determined by immunoblotting. (B) Changes in ARG1 palmitoylation levels induced by the C303A mutant were assessed using the ABE assay coupled with immunoblotting. (C) dHL‐60 cells were transfected with overexpressed FLAG‐tagged ARG1‐WT and FLAG‐ARG1‐C303A mutant. Anti‐FLAG immunoprecipitation was performed to enrich exogenous ARG1 protein, excluding signals derived from endogenous ARG1, followed by palmitoylation assessment via click chemistry. Representative immunoblot images are shown. (D) dHL‐60 cells overexpressing wild‐type ARG1 or the C303A mutant were treated with cycloheximide (CHX). Protein lysates were collected at the indicated time points, then ARG1 protein was monitored by immunoblotting. This experiment was repeated in triplicate independently with similar results. (E) The effects of 2‐BP on the degradation of ARG1 in dHL‐60 cells were measured by CHX‐chase assay under the condition of inhibitors for lysosome (Chloroquine), autophagy (3‐MA), proteasome (MG132) and caspase (DEVD‐CHO) treatment (left). Quantification of the relative ARG1 intensity (right). (F) CHX‐chase assay to display the degradation of wild‐type (WT) ARG1 or the C303A mutant in dHL‐60 cells treated with the inhibitors described in (E) (left). Quantification of the relative ARG1 intensity (right). (G) Immunoblot analysis of ARG1 in dHL‐60 cells with or without ZDHHC12 and ZDHHC18 siRNA transfection. Anti‐tubulin was used as a loading control. (H) Palmitoylated ARG1 levels in dHL‐60 cells with ZDHHC12 or ZDHHC18 knockdown were detected by ABE assay combined with immunoblotting. (I) Differentiated dHL‐60 cells were treated with or without SLC25A20 knockout tumor supernatant, palmitic acid, ZDHHC18‐siRNA or Palmostatin B for 48 h. Cells were collected for immunoblot‐based detection of ARG1 expression. (J) Representative immunofluorescence micrographs showing co‑localization of ZDHHC18 and ARG1 in TANs from human CRC tissues. (K) The physical interaction between ARG1 and ZDHHC18 in dHL‐60 cells was verified by Co‐IP coupled with Immunoblot analysis. (L) dHL‐60 cells were incubated with or without palmitic acid or 2BP for 48h, followed by Co‐IP and immunoblotting. The immunoblots in A‐I and K‐L are representative of three independent experiments. Original uncropped and unaltered blots for this panel are provided in Figure S16.

Given that the loss of palmitoylation compromises ARG1 stability, we further investigated the degradation pathways responsible for turnover of palmitoylation‐deficient ARG1 protein. We first evaluated classical protein degradation machinery using pharmacological inhibitors in dHL‐60 cells. Notably, the 2‐BP‐induced degradation of ARG1 was effectively rescued only by the lysosomal inhibitor chloroquine. By contrast, inhibitors targeting the proteasome (MG132), caspase (DEVD‐CHO), or autophagy (3‐MA) failed to restore ARG1 levels (Figure 6E). Consistently, chloroquine administration, but not other pathway inhibitors, successfully prevented the accelerated degradation of the ARG1‐C303A mutant (Figure 6F). These data indicate that ARG1 palmitoylation promotes protein stability by preventing lysosomal degradation.

Protein palmitoylation is catalyzed by a family of 23 human zinc finger DHHC‐type palmitoyl transferases (PATs, ZDHHC1‐23) [41]. To identify the specific PAT responsible for ARG1 palmitoylation in TANs, we analyzed PAT expression profiles using the Human Protein Atlas database (www.proteinatlas.org) and found that ZDHHC18 and ZDHHC12 were highly expressed in neutrophils (Figure S9A). To systematically identify ARG1‐interacting partners in neutrophils, we performed immunoprecipitation coupled with mass‐spectrometry (IP‐MS) in dHL‐60 cells. Among the candidate binding proteins, ZDHHC18 was recovered as one of the high‐confidence ARG1‐associated hits (Figure S9B). Furthermore, we performed siRNA‐mediated knockdown of ZDHHC18 or ZDHHC12 in both dHL‐60 cells and THP‐1‐derived macrophages to compare their regulatory roles across distinct lineages (Figure S9C and D). Interestingly, ZDHHC18 silencing specifically diminished ARG1 protein abundance without affecting its mRNA transcription in dHL‐60 cells (Figure 6G and Figure S10A), whereas this intervention exerted no discernible effect on THP‐1‐derived macrophages (Figure S10B). Notably, ABE assays confirmed that ZDHHC18 mediated palmitic acid‐induced ARG1 palmitoylation exclusively in neutrophils, with no parallel effect observed in macrophages (Figure 6H and Figure S10C). Moreover, ZDHHC18 silencing abrogated the induction of ARG1 expression triggered by SLC25A20‐depleted TCM, exogenous palmitic acid, or Palmostatin B (Figure 6I). Immunofluorescence staining further verified prominent colocalization of endogenous ARG1 and ZDHHC18 proteins in dHL‐60 cells (Figure 6J). Coimmunoprecipitation analyses confirmed a physical interaction between ARG1 and ZDHHC18 in dHL‐60 cells and macrophages (Figure 6K and Figure S10D). Intriguingly, the addition of palmitic acid strengthened this ARG1‐ZDHHC18 interaction in dHL‐60 cells, whereas treatment with 2‐BP disrupted it, with no parallel effect observed in macrophages (Figure 6L and Figure S10E). In summary, ZDHHC18 was specifically responsible for ARG1 palmitoylation in neutrophils but not in macrophages.

### 2.7 ARG1‐Palmitoylated Neutrophils Remodel the Immunosuppressive TME by Inhibiting CD8^+^ T Cell Proliferation to Promote CRC Progression

Given that a hallmark of immunosuppressive TANs is the suppression of antitumor immunity, we evaluated the impact of these functionally reprogrammed TANs on T cell proliferation. Compared to control conditions, dHL‐60 cells primed with TCM from SLC25A20‐depleted CRC cells significantly suppressed the proliferation of cocultured CD8^+^ T cells. Notably, the suppressive effect was reversed upon 2‐BP treatment or ZDHHC18 silencing (Figure 7A). This T cell‐dependent mechanism provides a compelling rationale for our earlier observation that SLC25A20‐KO did not distinctly alter the tumorigenic potential of CRC cells in T cell‐deficient nude mice. To further delineate the impact of this immunosuppressive axis on tumor progression, we established a tripartite in vitro coculture model comprising CRC cells, conditioned dHL‐60 cells and CD8^+^ T cells. Following 48 h of coculture, CRC cells were isolated to assess their biological properties. We observed that dHL‐60 cells preconditioned with TCM from SLC25A20‐depleted CRC cells potently blunted the antitumor cytotoxicity of CD8^+^ T cells, which consequently resulted in enhanced proliferation and colony formation ability of CRC cells. These effects were abrogated by 2‐BP treatment or ZDHHC18 silencing in dHL‐60 cells (Figure 7B and Figure S11A and B).

**FIGURE 7:**
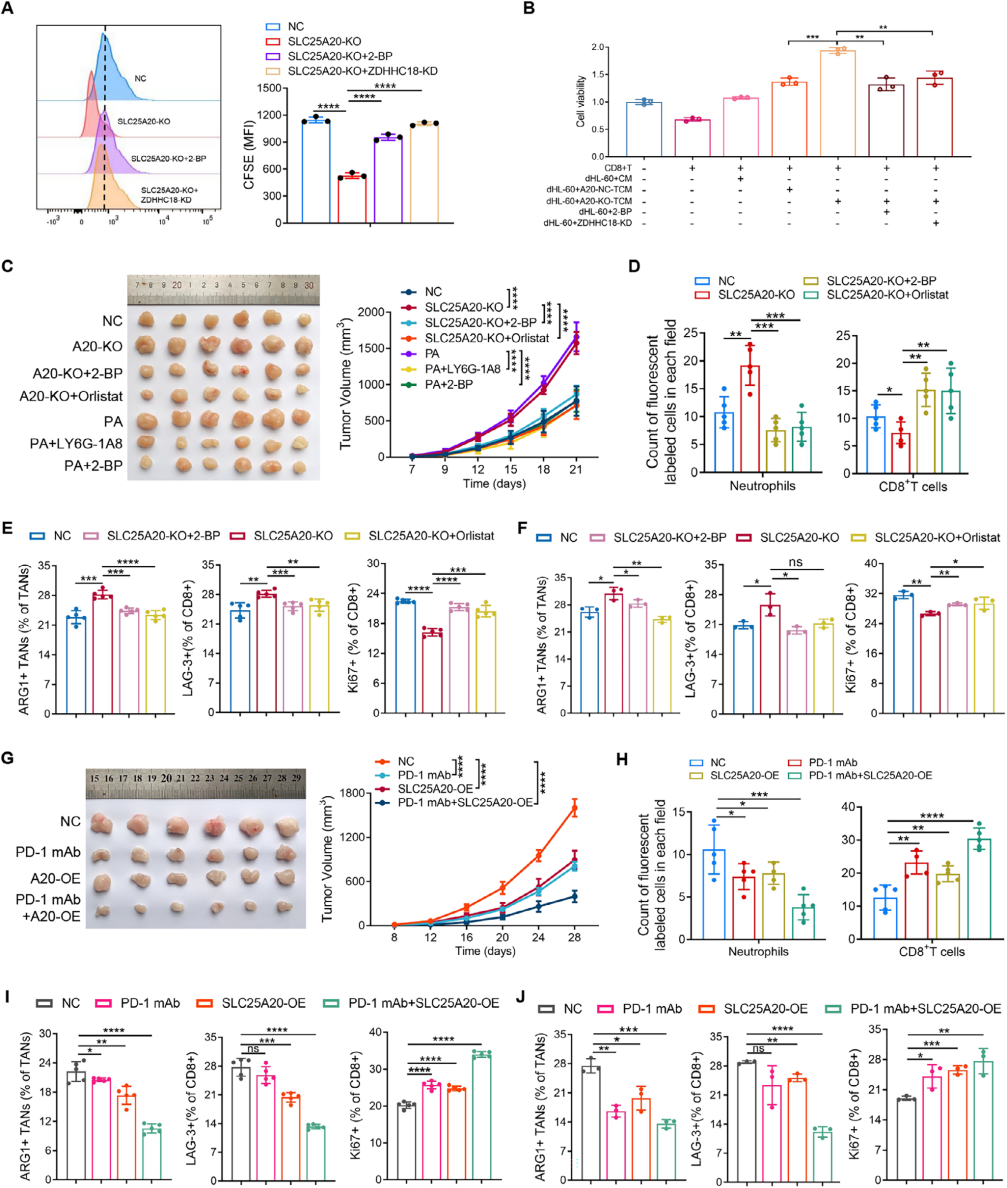
ARG1‐palmitoylated neutrophils drive immunosuppression and facilitate CRC progression by restraining CD8^+^T‐cell antitumor responses. (A) CFSE was used to demonstrate the effect of dHL‐60 cells domesticated under different conditions on the proliferation ability of CD8^+^ T cells (n = 3). (B) CCK‐8 assays were performed to evaluate the proliferation of tumor cells from dHL‐60‐CD8^+^ T‐CRC cell co‐culture system (n = 3). (C) Representative images of subcutaneous tumors (left) and growth curves of tumor volume (right) obtained from C57BL/6 mice subcutaneously injected with SLC25A20 knockout or control MC38 cells (n = 6 per group). (D) Statistical analysis of SLC25A20, Ly6G, CD8 and DAPI‐based quantitative fluorescence signals in subcutaneous tumor tissues from C57BL/6 mice bearing SLC25A20‐KO tumors, with or without 2‐BP or Orlistat treatment (n = 5 per group). (E and F) Flow cytometry‐based analysis of specific molecule expression on TANs and CD8^+^ T cells infiltrating subcutaneous tumors (n = 5) or cecal orthotopic tumors (n = 3). Cells were isolated from SLC25A20‐KO and control MC38‐bearing C57BL/6 mice, with or without 2‐BP and Orlistat treatment. (G) Representative images of subcutaneous tumors (left) and growth curves of tumor volume (right) obtained from MC38 tumor‐bearing mice treated with an isotype control, an anti‐PD‐1 mAb, an SLC25A20 overexpression treatment or the anti‐PD‐1 mAb combined with SLC25A20 overexpression treatment (n = 6 per group). (H) Quantitative statistical analysis of fluorescence signals for SLC25A20, Ly6G, CD8, and DAPI in subcutaneous tumors from MC38‐bearing mice receiving isotype control, anti‐PD‐1 mAb, SLC25A20 overexpression, or combined anti‐PD‐1 mAb and SLC25A20 overexpression treatment (n = 5 per group). (I and J) Flow cytometry‐based analysis of specific molecule expression on TANs and CD8^+^ T cells infiltrating subcutaneous tumors (n = 5) or cecal orthotopic tumors (n = 3). Cells were isolated from MC38‐bearing C57BL/6 mice receiving anti‐PD‐1 mAb, SLC25A20 overexpression, or combined anti‐PD‐1 mAb and SLC25A20 overexpression treatment. ns denotes nonsignificant. Data are presented as the mean ± SD. Statistical significance was calculated by one‐way ANOVA with Tukey's post hoc test (A‐B, D‐F), one‐way ANOVA with Dunnett's post hoc test (H‐J) and by two‐way ANOVA (C and G). *P < 0.05, ^**^ *P* < 0.01, ^***^ *P* < 0.001, and ^****^ *P* < 0.0001.

Furthermore, we proposed that disrupting palmitoylation or FA synthesis may inhibit CRC growth in vivo. As anticipated, intraperitoneal administration of 2‐BP or Orlistat in MC38 tumor‐bearing C57BL/6 mice effectively abrogated the tumor‐promoting effects of SLC25A20 KO, restricting tumor growth to levels comparable to those of the control group (Figure 7C). Consistent with this, 2‐BP or Orlistat treatment significantly attenuated the SLC25A20 KO‐driven alterations in the TME, specifically limiting TAN infiltration and rescuing the concurrent CD8^+^ T cell exclusion (Figure 7D and Figure S12A). Similar to the effects of SLC25A20 deficiency, the administration of exogenous palmitic acid accelerated CRC growth, and this acceleration was abrogated either by neutrophil depletion using LY6G‐1A8 antibody or 2‐BP treatment (Figure 7C). Furthermore, SLC25A20‐KO tumors exhibited an expanded population of tumor‐infiltrating ARG1^+^ neutrophils, accompanied by elevated expression of exhaustion markers (PD‐1, LAG‐3, and CTLA‐4) and diminished expression of proliferation marker Ki67 in CD8^+^ T cells (Figure 7E and Figure S12B). Notably, therapeutic intervention with either 2‐BP or Orlistat effectively reversed these effects driven by SLC25A20 deficiency (Figure 7E and Figure S12B). In addition, we employed an orthotopic CRC mouse model, which better recapitulates the native tumor microenvironment of CRC, and obtained experimental results consistent with subcutaneous tumor‐bearing mice (Figure 7F and Figure S12C).

Given that CD8^+^ T cells are critical effectors targeted by immune checkpoint blockade (ICB), we hypothesized that restoring SLC25A20 expression might synergize with anti‐PD‐1 treatment. As anticipated, either ectopic SLC25A20 overexpression in MC38 cells or anti‐PD‐1 monotherapy significantly hindered tumor growth (Figure 7G). Both individual interventions also effectively restricted the infiltration of TANs while promoting CD8^+^ T cell recruitment (Figure 7H and Figure S13A). Impressively, the combination therapy elicited a robust synergistic response, leading to a profound suppression of tumor progression (Figure 7G), and further remodeling the intratumoral immune landscape (Figure 7H and Figure S13A). Flow cytometry confirmed that the combination therapy significantly diminished the population of ARG1^+^ neutrophils, mitigated T cell exhaustion and reinvigorated CD8^+^ T cell proliferation within the TME (Figure 7I and Figure S13B). The orthotopic CRC mouse model also confirmed this result (Figure 7J and Figure S13C). Collectively, these results suggest that the restoring SLC25A20 effectively reinvigorates T‐cell‐mediated antitumor immunity, highlighting its potential translational value in combination with ICB.

## 3 Discussion

Metabolic reprogramming, particularly enhanced lipid metabolism, is a recognized hallmark of various malignancies [17, 42]. However, the precise mechanisms by which cancer cells dynamically recalibrate lipid homeostasis to adapt to TME stresses remain incompletely understood. In this study, we identified the downregulation of SLC25A20 as a pivotal driver of CRC progression and poor prognosis of CRC patients, operating primarily through the orchestration of an immunosuppressive TME. Notably, the prognostic benefit of SLC25A20 was restricted to advanced‐stage CRC in our IHC cohort, yet this stage‐specific effect was not recapitulated in TCGA‐COAD/READ, highlighting cohort‐dependent prognostic value that awaits further validation. Mechanistically, SLC25A20 deficiency in tumor cells impairs mitochondrial LCFA transport, thereby blunting FAO and triggering a compensatory efflux of palmitic acid‐enriched lipids into the TME. In turn, these tumor‐derived palmitic acids are internalized by TANs, wherein they are utilized as substrates to enhance ZDHHC18‐mediated ARG1 palmitoylation. This specific PTM drastically augments ARG1 protein stability. The consequent accumulation of ARG1 within TANs dampens CD8^+^ T cell proliferation and abrogates antitumor immunity, ultimately accelerating CRC progression (Figure 8).

**FIGURE 8:**
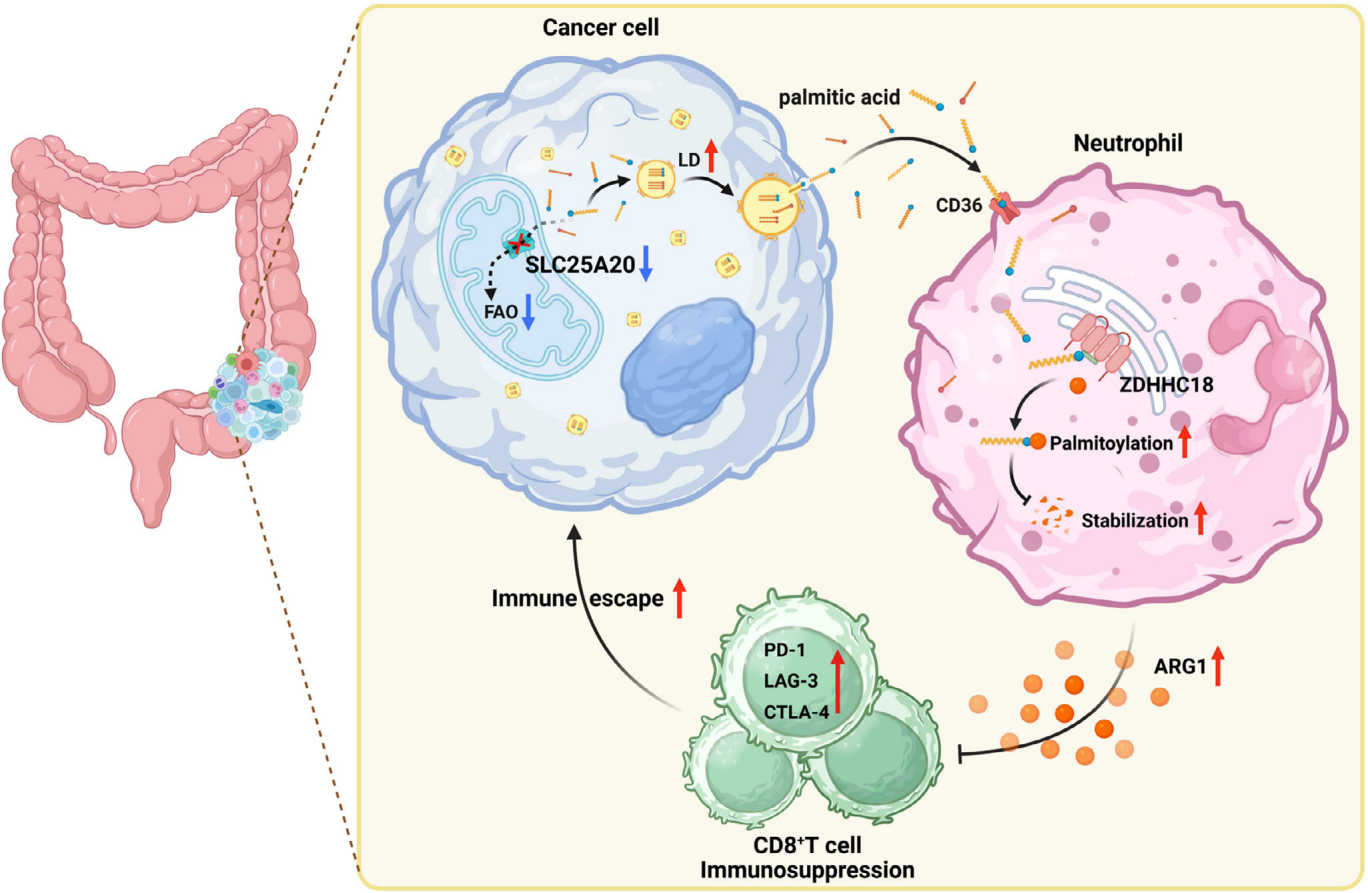
Schematic illustration displaying the mechanism of action of SLC25A20 in CRC. In CRC cells, SLC25A20 deficiency impairs mitochondrial LCFA transport and decreases FAO, which results in increased palmitic acid‐enriched FA release into the TME. These palmitic acids are internalized by TANs, where ARG1 palmitoylation is mediated by ZDHHC18, enhancing its protein stability. This process contributes to suppressing CD8^+^ T cell function and promotes CRC progression. The illustration was created with BioRender (available online: https://biorender.com/).

Rapidly proliferating tumor cells exhibit a profound reliance on lipid metabolism [43, 44]. While the increased lipogenesis is a well‐established oncogenic hallmark, the precise role of FAO in cancer remains highly context‐dependent. Although enhanced FAO can fuel cancer cell survival, excessive flux may induce lethal oxidative stress [20, 21]. Furthermore, FAO and lipogenesis are mutually exclusive processes reciprocally modulated by malonyl‐CoA [45], an intermediate of FA biosynthesis and a potent allosteric inhibitor of CPT1, implying that FAO activation is not universally required for oncogenesis. Consistent with this metabolic heterogeneity, our results demonstrated that SLC25A20 deficiency accelerated CRC growth in vivo, inherently linked to the impaired mitochondrial entry of LCFAs and the subsequent blockade of FAO. This tumor‐promoting effect of compromised FAO aligns with observations in HCC, where SLC25A20 downregulation similarly promotes HCC growth and metastasis [23]. Corroborating this, another study revealed that the downregulation of SLC25A20 and CPT2 limits FAO and causes acylcarnitine accumulation, which paradoxically attenuates lipotoxicity and facilitates hepatocarcinogenesis in high‐fat diet (HFD)‐driven models [22]. Conversely, a more recent study in pancreatic ductal adenocarcinoma (PDAC) reported an opposing phenotype: SLC25A20 knockdown inhibited cancer growth by depleting ATP production and inactivating mTOR, culminating in cancer cell death [46]. Together, these divergent observations underscore that the FAO pathway is dynamically coupled to context‐specific oncogenic or tumor‐suppressive events. Therefore, targeted therapeutic strategies directed at specific metabolic enzymes must be meticulously tailored to the needs of each cancer type.

While the intracellular accumulation of unoxidized FAs typically incites lethal lipotoxicity [47], SLC25A20‐deficient CRC cells remarkably circumvent this metabolic stress by channeling excess lipids into LDs and augmenting free FA efflux. This dynamic lipid rewiring extends beyond fueling tumor cells; it actively remodels the immune microenvironment to foster a protumorigenic TIME [39, 48]. Previous studies have suggested that tumor‐derived lipids, such as LCFAs and cholesterol metabolites, can dictate the polarization of TAMs toward an M2 phenotype or recruit immunosuppressive myeloid cells to facilitate metastasis [32, 49]. Importantly, we also observed that SLC25A20‐downregulated CRC cells released abundant free FAs into the TME and recruited TANs. Although our data suggest intact LD turnover is required for elevated FFA release, strict causality cannot be confirmed here. Conventional isotope tracing fails to separate directly secreted FFAs from LD‐derived FFAs. Rigorous validation will demand technically demanding pulse‐chase tracing combined with LD fractionation in future studies.

As a key component of the TME, TANs exhibit phenotypic plasticity and are increasingly recognized as primary drivers of immunosuppression, therapy resistance, and adverse clinical outcomes [14, 50, 51]. Notably, functional depletion of TANs using an anti‐Ly6G antibody (clone 1A8) completely abrogated the accelerated in vivo tumor growth induced by SLC25A20 deficiency. Collectively, these findings delineate a novel metabolic‐immune crosstalk axis, establishing SLC25A20‐mediated lipid efflux as a pivotal regulator of intercellular communication between CRC cells and neutrophils. Although the peripheral NLR serves as an accessible systemic biomarker, it cannot fully capture the complex intratumoral immune landscape. By integrating NLR with comprehensive intratumoral immune profiling (Figure 2), our findings indicate that SLC25A20 modulates anti‐tumor immunity primarily by regulating local neutrophil infiltration, highlighting the necessity of assessing intratumoral immune dynamics alongside systemic indicators.

Beyond serving as alternative energetic fuels, accumulating metabolites also regulates gene transcription, epigenetic regulation, and PTMs [39]. As the primary substrate for protein palmitoylation, palmitic acid remodels the dynamic landscape of immune regulatory factors [52, 53]. Although ARG1 is a classical hallmark of immunosuppressive myeloid cells, which dampens T cell activation by local arginine depletion [54, 55, 56], its regulation has mainly been attributed to transcriptional control. Here, we revealed a novel lipid‐driven PTM mechanism whereby tumor‐derived palmitate directly dictates TAN function through ARG1 stabilization. Notably, we identified a previously unrecognized ZDHHC18‐catalyzed palmitoylation of ARG1 at Cys303, which protects ARG1 protein from degradation. Palmitoylation studies rely on two complementary strategies: global acyl‐proteomic profiling and targeted‐site validation. Global acyl‐proteomic allows unbiased substrate screening, whereas targeted assays permit precise verification of individual cysteine sites. Here, we employed targeted validation to confirm the functional Cys303 palmitoylation of ARG1. Future studies should combine both approaches to fully decipher palmitoylation‐dependent regulation in tumor‐immune metabolism. In addition, we verified that palmitoylation shields ARG1 from lysosomal, rather than proteasomal, degradation. The Cys303 mutation accelerated ARG1 turnover, which was rescued by chloroquine but not MG132. Because non‐proteasomal degradation often relies on ubiquitin sorting signals, especially K63‐linked polyubiquitination [57], depalmitoylation may unmask specific degrons for E3 ubiquitin ligase recognition. The specific ubiquitination‐related enzymes involved in ARG1 turnover remain undefined and require further exploration in future work. Simultaneously, palmitoylation is a dynamically reversible PTM coordinated by ZDHHC palmitoyltransferases and depalmitoylases [58]. Palmostatin‐B‐mediated pan‐depalmitoylase inhibition increased ARG1 levels in dHL‐60 cells, validating a dynamically reversible cycle. Failure to identify the exact depalmitoylase isoform is a limitation of the present study, future systematic mass‐spectrometry‐based profiling will help complete this regulatory circuit. Our findings reveal that the SLC25A20‐palmitic acid‐ZDHHC18‐ARG1 cascade serves as a molecular bridge connecting tumor lipid metabolism to TAN‐mediated immunosuppression in CRC, representing a major rather than exclusive pathway for immunosuppressive TAN remodeling. While lipidomic profiling identified palmitic acid as the most significantly upregulated free LCFA upon SLC25A20 depletion, other accumulated lipid species and soluble metabolites within the TME may also contribute to immune modulation. Similarly, tumor‐secreted chemokines, TGF‐β and inflammatory cytokines may act in parallel or synergistically to modulate TAN phenotype. TGF‐β upregulation is not obligately induced by SLC25A20 loss but can emerge under severe lipotoxic stress. Future systematic screening is required to dissect the relative contributions and potential synergy between lipid metabolites and secreted protein mediators in shaping the TIME.

We evaluated the interventional effects by pharmacologically disrupting this lipid‐driven PTM regulatory axis. Unlike 2‐BP, which directly blocks palmitoyltransferases, Orlistat is a broad‐spectrum covalent inhibitor targeting serine hydrolase family members, encompassing fatty‐acid synthase (FASN) as well as intracellular neutral lipases ATGL and HSL [59, 60]. In our tumor model, Orlistat was employed to indirectly impair ARG1 palmitoylation via substrate depletion by restricting tumor de novo fatty acid synthesis and LD catabolism, limiting free palmitate liberation and its abundance in the TME. Such substrate depletion lowers the cellular acyl‐CoA pool accessible for ZDHHC18‐dependent ARG1 palmitoylation and consequently interrupts the downstream pro‐tumorigenic signaling cascade. To circumvent Orlistat's classical gastrointestinal limitations, we utilized intraperitoneal injection which enabled Orlistat to act directly as a FASN inhibitor against tumor‐intrinsic lipogenesis in mice, diminishing the extracellular palmitate pool available for TAN uptake and subsequent ZDHHC18 catalysis. Nevertheless, Orlistat lacks absolute FASN selectivity and may inhibit other serine hydrolases, meaning potential systemic off‐target effects in vivo cannot be entirely ruled out. Future studies utilizing highly selective FASN inhibitors will help address these limitations and further validate this metabolic axis.

Although our mechanistic focus centers on TANs, SLC25A20‐mediated immune regulation is fully immune‐dependent and may involve coordinated alterations in multiple immune compartments. Our findings further demonstrate that this metabolic‐immune crosstalk operates via a dominant neutrophil‐CD8^+^ T cell axis. Functionally, ARG1‐mediated immunosuppressive TANs trigger CD8^+^ T cell exhaustion, characterized by impaired proliferation and elevated expression of PD‐1, LAG‐3, and CTLA‐4. Notably, the pro‐tumorigenic effect of SLC25A20 deficiency was fully abrogated upon neutrophil depletion or in T cell‐deficient mice, confirming the specificity of this immunosuppressive cascade. Importantly, both human and mouse profiling revealed that macrophage infiltration remained unaltered regardless of SLC25A20 status. Parallel functional assays in THP‐1‐derived macrophages corroborated that the SLC25A20‐palmitate‐ZDHHC18‐ARG1 axis was restricted to TANs rather than broadly active across all myeloid lineages. However, distinct immune cell populations display divergent lipid uptake capabilities and palmitoylation‑dependent signaling responses. Future comprehensive immune profiling will help clarify how SLC25A20‑mediated lipid rewiring modulates other TME components and additional immune subsets.

Despite the promise of ICB in oncology, clinical responses in CRC remain generally poor due to substantial TME immunosuppression [61]. Here, we demonstrate that restoring SLC25A20 expression synergizes potently with anti‐PD‐1 therapy, even in a lipid‐rich environment. This combination markedly depleted immunosuppressive ARG1^+^ TANs, attenuated CD8^+^ T cell exhaustion, and restored intratumoral CD8^+^ T cell proliferation. While our study identifies the SLC25A20‐palmitate‐ZDHHC18‐ARG1 cascade as a primary therapeutic target, the precise molecular mechanisms by which SLC25A20‐mediated neutrophil remodeling enhances PD‐1 blockade responsiveness remain to be fully elucidated in future investigations.

Overall, our findings confirm that SLC25A20 is a functional marker of CRC that restrains tumor progression by altering the phenotype of neutrophils and inhibiting the metastatic capacity of cancer cells. Moreover, we emphasize that palmitoylation mediated by palmitic acid is the primary pathway involved in remodeling the neutrophil phenotype. This study not only provides insight into the biological function of SLC25A20 but also offers important theoretical and experimental evidence for effective control of the malignant process of CRC.

## 4 Methods

### 4.1 Clinical Specimens

The use of human tissue specimens for this study was approved by the ethics committee of Nanfang Hospital, Southern Medical University (Guangzhou, China; No. NFEC‐2025‐422). All of the patients signed an informed consent form before their clinical materials were used for research purposes. Fresh and paraffin‐embedded CRC tissue samples were obtained from patients diagnosed with primary CRC at Nanfang Hospital, Southern Medical University. A total of 32 fresh CRC cases and their paired nontumor mucosal tissues were stored in liquid nitrogen for later use. And a cohort of 113 paraffin tissues via IHC analysis to compare the expression of SLC25A20.

### 4.2 Construction of Cell Lines with Stable Overexpression or Knockout of SLC25A20

The stable CRC cell lines were obtained by SLC25A20 overexpression or control lentivirus transduction and screening with puromycin. CRISPR‐Cas9 technology was used to construct stable SLC25A20 knockout CRC cell lines. The lentivirus primers and the gRNA sequences required for CRISPR‐Cas9 were listed in Table S1.

#### 4.2.1 RNA Isolation and Real‐Time PCR (RT‐PCR)

Total RNA in tissues and cells was obtained by Trizol reagent (TaKaRa, Dalian, China). The synthesis of cDNA was carried out using the Prime Script RT (TaKaRa) assay kit. RT‐PCR was performed thought SYBR Premium Ex Taq II (TaKaRa). All results were normalized to the GAPDH expression. Each sample was tested in triplicate to eliminate technical variability as much as possible. The primer sequences were listed in Table S2.

### 4.3 Oligonucleotide Transfection

The siRNA DGAT1, siRNA DGAT2, siRNA ATGL, siRNA CD36, siRNA ZDHHC18, siRNA ZDHHC12 and negative control siRNA were synthesized from GenePharma (Shanghai, China). The cells transfection according to the manufacturer's instructions of Lipo3000. All si‐RNAs sequences were listed in Table S3.

### 4.4 Functional Assays in Vitro and in Vivo

The in vitro cell proliferation and colony formation experiments, as well as the in vivo subcutaneous tumorigenesis and metastasis assays were conducted according to our previously published experimental protocols [62]. More details were supplied in the Supplemental Methods.

### 4.5 ARG1 Mutant Construction

The vectors encoding Flag‐ARG1 were synthesized by inserting complementary DNAs into the pcDNA3.1 (RRID: Addgene_100544) vector. The site‐specific mutagenic PCR reaction was applied to generate mutants of ARG1 C303A. Finally, sequencing was performed to confirm whether the construction was successfully. The sequences of primers used for ARG1 mutant were listed in Table S4.

### 4.6 Acyl‐Biotin Exchange (ABE) Experiment

The ABE assay according to Azhar Ali and colleagues, simultaneously making certain modifications [63]. First, dHL‐60 cells from each treatment group were collected and lysed at 4 °C for 30 min using lysis buffer (LB pH 7.4) containing N‐ethylmaleimide (NEM, E3876, Sigma), 50 mM Tris‐HCl, 150 mM NaCl, 1% NP‐40, 1 mM EDTA, and protease inhibitors. Then, three times consecutive methanol chloroform (CM) precipitates. The samples were co incubated with ARG1 antibody overnight at 4 °C. Subsequently, Protein G Sepharose 4 beads (#17‐0618‐02, GE Healthcare) were used for incubating samples at room temperature for 2 h. After centrifugation at 8200 g for 30 s and 600 µL Stringent Buffer was added, then 300 µL was transferred to 1 ml hydroxylamine (HAM 1M, #379921, Sigma) Buffer (HA + group) as the experimental group, and the residual 300 µL was added into 1ml HAM free buffer as the control group (HA‐ group), continuous flipping at room temperature for 2 h. HAM Buffer to the HA+ group, and LB to the HA‐ group, continuous incubation at room temperature for 1 h. After being rinsed by LB for three times, followed by treatment with BMCC‐Biotin (#21900, Thermo Fischer Scientific) for 2 h at room temperature with soft rotation. Finally, the obtained samples were eluted by LB and analyzed using SDS‐PAGE.

### 4.7 Fatty Acid Oxidation Assay

The FAO level was detected by the FAO Kit following the manufacturer's instructions [64]. Cells were seeded into a culture dish containing Geltrex coating. After 24 h, the cells were washed twice with cold PBS and resuspended in PBS. A total of 1.5 × 10^6^ cells were centrifuged under 200 g conditions for 5 min, then adding 70 µL lysis buffer, followed by a centrifugation at 12,000 rpm for 5 min. Following the combination of 10 µL sample with 50 µL reaction solution in a 96‐well plate, the mixture was incubated at 37 °C (free of CO_2_) for 2 h. The entire reaction was stopped by 50 µL freshly configured ice acetic acid (3%), and measured the absorbance value (OD value) at a wavelength of 492 nm.

### 4.8 IHC Analysis

After incubating overnight with primary antibodies anti‐SLC25A20 (RRID: AB_10642001, 1:200), anti‐CD66b (RRID: AB_2077866, 1:200), anti‐CD8, and anti‐CD68 at 4 °C, Paraffin sections were co incubated with secondary antibodies at room temperature for 1 h. Diaminobenzidine (DAB; ZLI‐9018) processing at the same time was used for color rendering. Finally, using hematoxylin to complete the staining of the nucleus. All antibodies used for IHC were listed in Table S5.

Following the principle of blinding, 3 pathologists were selected to evaluate the staining results. According to the staining results, it was preliminarily divided into negative signal (colorless) and positive signal (yellow). Positive signals were graded based on different coloring intensities: 0 (no coloring) ‐1 (light yellow) ‐2 (yellow) ‐3 (brown yellow) ‐4 (brown). In addition, the extent of staining, based on the percentage of coloring area, set a rating score of 0 (0%) ‐1 (1%–25%) ‐2 (26%–50%) ‐3 (51%–75%) ‐4 (76%–100%). The final staining score for SLC25A20 was calculated by the summation of the staining‐extent and staining‐intensity scores. The final staining score ≥ 3 was defined as high SLC25A20 expression, while < 3 was defined as low expression.

### 4.9 Metabolomics Analysis

The culture supernatant was collected from each group of cells. All samples were analyzed for FFA in absolute scale (µM/L) by the mass spectrum method [65].

### 4.10 Multiple Immunofluorescence Staining

The paraffin tissue sections were subjected to an IHC procedure, where the first antibody was incubated with SLC25A20 (RRID: AB_10642001, 1:100) until the end of Polymer HRP secondary antibody incubation. Subsequently, TSA dye was dripped and incubated at room temperature in the dark for 10 min, followed by 1 × TBST solution (55 ml TBST + 1000 ml high‐pressure sterilized water + 1 ml Tween‐20 + 300 µL TritonX‐100), repeated the steps of antigen repair until blockade, and incubated the remaining primary antibodies in sequence (TIP47, RRID: AB_881572,1:250; CD66b, RRID: AB_2077866, 1:150; ARG1, RRID: AB_2289842, 1:100; CD68, 1:200; CD8, 1:200; LY6G, RRID: AB_2923218, 1:500; ZDHHC18, RRID: AB_2762091, 1:50) until all the target antibodies dyeing completed. After TBST washing, DAPI was dripped at room temperature in the dark for 10 min to stain the nucleus, sealing of slices with anti‐quenching reagents, and with an observation by the full‐automatic digital section scanning system (KF‐PRO‐005). All antibodies used for multiple immunofluorescence staining were listed in Table S5.

#### 4.10.1 Coimmunoprecipitation (CoIP)

dHL‐60 cells with different treatments were lysed in IP buffer (containing 20 mM Tris‐HCl, pH 8.0, 100 mM NaCl, 2 mM EDTA and 1% Nonidet P‐40) supplemented with 1 mM PMSF and a protease inhibitor cocktail. ARG1 (RRID: AB_2289842) or ZDHHC18 (RRID: AB_2762091) antibody was applied to the cell lysates and incubated at 4 °C overnight. The samples were immunoprecipitated with 50 µL of magnetic beads at 4 °C overnight. The beads were washed three times with cold lysis buffer, then boiled at 100 °C for 5 min and analyzed by Western blotting. All antibodies used for CoIP were listed in Table S5.

Other procedures protocols were supplied in the Materials and Methods.

### 4.11 Statistics

All experimental data were presented as mean ± standard deviation. All statistical analyses were performed using SPSS Statistics version 22 (IBM Corp., Armonk, NY, USA). Comparisons between two groups were made using unpaired two‐tailed Student's t‐tests, while one‐way or two‐way ANOVA followed by Tukey's post hoc test was used for comparisons among multiple groups. Survival curves were plotted using the Kaplan‐Meier method and compared by the log‐rank test. *P* < 0.05 was defined as statistically significant.

Sample sizes were not predetermined by power analysis but were based on prior experimental experience and relevant literature. Sample sizes and replicates were detailed in the corresponding figure legends. No data points were excluded from the analyses.

## Author Contributions

Conceptualization: **Y.H**., **S.W**.; Methodology: **S.W**., **Y.H**., **T.T**.; Investigation: **Y.H**., **T.T**., **B.Z**., **Y.F**., **W.C**., **M.Y**., **Y.W**.; Formal analysis: **H.Y**., **T.T**., **S.W**.; Writing – original draft: **Y.H**., **S.W**.; Writing – review & editing: **Y.H**., **S.W**.; Funding acquisition: **S.W**., **Y.D**., **Y.W**., **Y.H**.

## Funding

This work was supported by the National Natural Science Foundation of China (82473083, 82073237, and 82472777), the Guangdong Basic and Applied Basic Research Foundation (2025A1515010489) and China Postdoctoral Science Foundation (2024M761341).

## Conflicts of Interest

The authors declare no conflicts of interest.

## Supporting information




**Supporting File**: advs78114‐sup‐0001‐SuppMat.docx.

## Data Availability

The RNA‐sequencing data and clinical information of CRC samples were obtained from The Cancer Genome Atlas (TCGA; https://www.cancer.gov/tcga) via TCGAbiolinks R/Bioconductor package (version 2.2.0). All data supporting the findings of this study and its supplementary information files are available in the “supporting data values” file.

## References

[1] R. L. Siegel , K. D. Miller , N. S. Wagle , and A. Jemal , “Cancer statistics,” CA: A Cancer Journal for Clinicians 73, no. 1 (2023): 17–48. 10.3322/caac.21763 36633525

[2] C. C. Murphy and T. A. Zaki , “Changing epidemiology of colorectal cancer — birth cohort effects and emerging risk factors,” Nature Reviews Gastroenterology & Hepatology 21, no. 1 (2024): 25–34. 10.1038/s41575-023-00841-9 37723270

[3] R. L. Siegel , K. D. Miller , S. A. Fedewa , et al., “Colorectal cancer statistics,” CA: A Cancer Journal for Clinicians 70, no. 3 (2020): 145–164. 10.3322/caac.21601 32133645

[4] M. Sadeghi , S. Dehnavi , M. Sharifat , A. M. Amiri , and A. Khodadadi , “Innate Immune Cells: Key Players of Orchestra in Modulating Tumor Microenvironment (TME),” Heliyon 10, no. 5 (2024): e27480. 10.1016/j.heliyon.2024.e27480 PMC10923864 38463798

[5] K. E. de Visser and J. A. Joyce , “The evolving tumor microenvironment: From cancer initiation to metastatic outgrowth,” Cancer Cell 41, no. 3 (2023): 374–403. 10.1016/j.ccell.2023.02.016 36917948

[6] D. Hanahan and R. A. Weinberg , “Hallmarks of cancer: The next generation,” Cell 144, no. 5 (2011): 646–674. 10.1016/j.cell.2011.02.013 21376230

[7] S. B. Coffelt , M. D. Wellenstein , and K. E. Visser , “Neutrophils in cancer: Neutral no more,” Nature Reviews Cancer 16, no. 7 (2016): 431–446. 10.1038/nrc.2016.52 27282249

[8] S. Jaillon , A. Ponzetta , D. Di Mitri , A. Santoni , R. Bonecchi , and A. Mantovani , “Neutrophil diversity and plasticity in tumour progression and therapy,” Nature Reviews Cancer 20, no. 9 (2020): 485–503. 10.1038/s41568-020-0281-y 32694624

[9] R. Xue , Q. Zhang , Q. Cao , et al., “Liver tumour immune microenvironment subtypes and neutrophil heterogeneity,” Nature 612, no. 7938 (2022): 141–147. 10.1038/s41586-022-05400-x 36352227

[10] Z. G. Fridlender , J. Sun , S. Kim , et al., “Polarization of Tumor‐Associated Neutrophil Phenotype by TGF‐β: “N1” versus “N2” TAN,” Cancer Cell 16, no. 3 (2009): 183–194. 10.1016/j.ccr.2009.06.017 PMC2754404 19732719

[11] B. B. Antuamwine , R. Bosnjakovic , F. Hofmann‐Vega , et al., “N1 versus N2 and PMN‐MDSC: A critical appraisal of current concepts on tumor‐associated neutrophils and new directions for human oncology,” Immunological Reviews 314, no. 1 (2023): 250–279. 10.1111/imr.13176 36504274

[12] V. Marteau , N. Nemati , K. Handler , et al., “Single‐cell integration and multi‐modal profiling reveals phenotypes and spatial organization of neutrophils in colorectal cancer,” Cancer Cell 44, no. 1 (2026): 146–165.e14. 10.1016/j.ccell.2025.12.003 41529665

[13] T. M. Bui , L. K. Yalom , E. Ning , et al., “Tissue‐specific reprogramming leads to angiogenic neutrophil specialization and tumor vascularization in colorectal cancer,” Journal of Clinical Investigation 134, no. 7 (2024): e174545. 10.1172/JCI174545 PMC10977994 38329810

[14] A. J. Gentles , A. M. Newman , C. L. Liu , et al., “The prognostic landscape of genes and infiltrating immune cells across human cancers,” Nature Medicine 21, no. 8 (2015): 938–945. 10.1038/nm.3909 PMC4852857 26193342

[15] T. M. Bui , V. Butin‐Israeli , H. L. Wiesolek , et al., “Neutrophils Alter DNA Repair Landscape to Impact Survival and Shape Distinct Therapeutic Phenotypes of Colorectal Cancer,” Gastroenterology 161, no. 1 (2021): 225–238.e15. 10.1053/j.gastro.2021.03.027 33753103

[16] J. A. Mosier , S. C. Schwager , D. A. Boyajian , and R.‐K. CA , “Cancer cell metabolic plasticity in migration and metastasis,” Clinical & Experimental Metastasis 38, no. 4 (2021): 343–359. 10.1007/s10585-021-10102-1 34076787

[17] M. Martin‐Perez , U. Urdiroz‐Urricelqui , C. Bigas , and S. A. Benitah , “The role of lipids in cancer progression and metastasis,” Cell Metabolism 34, no. 11 (2022): 1675–1699. 10.1016/j.cmet.2022.09.023 36261043

[18] L. A. Broadfield , A. A. Pane , A. Talebi , J. V. Swinnen , and S. M. Fendt , “Lipid metabolism in cancer: New perspectives and emerging mechanisms,” Developmental Cell 56, no. 10 (2021): 1363–1393. 10.1016/j.devcel.2021.04.013 33945792

[19] I. R. Schlaepfer and M. Joshi , “CPT1A‐mediated Fat Oxidation, Mechanisms, and Therapeutic Potential,” Endocrinology 161, no. 2 (2020): bqz046. 10.1210/endocr/bqz046 31900483

[20] Y. Xuan , H. Wang , M. M. Yung , et al., “SCD1/FADS2 fatty acid desaturases equipoise lipid metabolic activity and redox‐driven ferroptosis in ascites‐derived ovarian cancer cells,” Theranostics 12, no. 7 (2022): 3534–3552. 10.7150/thno.70194 PMC9065188 35547771

[21] T. Fukawa , B. C. Yan‐Jiang , J. C. Min‐Wen , et al., “Excessive fatty acid oxidation induces muscle atrophy in cancer cachexia,” Nature Medicine 22, no. 6 (2016): 666–671. 10.1038/nm.4093 27135739

[22] M. G. Schooneman , F. M. Vaz , S. M. Houten , and M. R. Soeters , “Acylcarnitines,” Diabetes 62, no. 1 (2013): 1–8. 10.2337/db12-0466 PMC3526046 23258903

[23] P. Yuan , J. Mu , Z. Wang , et al., “Down‐regulation of SLC25A20 promotes hepatocellular carcinoma growth and metastasis through suppression of fatty‐acid oxidation,” Cell Death & Disease 12, no. 4 (2021): 361. 10.1038/s41419-021-03648-1 PMC8024385 33824298

[24] Z. Jin , Y. D. Chai , and S. Hu , “Fatty Acid Metabolism and Cancer,” Advances in Experimental Medicine and Biology 1280 (2021): 231–241. 10.1007/978-3-030-51652-9_16 33791986

[25] N. Fujiwara , H. Nakagawa , K. Enooku , et al., “CPT2 downregulation adapts HCC to lipid‐rich environment and promotes carcinogenesis via acylcarnitine accumulation in obesity,” Gut 67, no. 8 (2018): 1493–1504. 10.1136/gutjnl-2017-315193 PMC6039238 29437870

[26] A. Valentino , A. Calarco , A. Di Salle , et al., “Deregulation of MicroRNAs mediated control of carnitine cycle in prostate cancer: Molecular basis and pathophysiological consequences,” Oncogene 36, no. 43 (2017): 6030–6040. 10.1038/onc.2017.216 28671672

[27] W. T. Kim , S. J. Yun , C. Yan , et al., “Metabolic Pathway Signatures Associated with Urinary Metabolite Biomarkers Differentiate Bladder Cancer Patients From Healthy Controls,” Yonsei Medical Journal 57, no. 4 (2016): 865–871. 10.3349/ymj.2016.57.4.865 PMC4951461 27189278

[28] V. Andersen , U. Halekoh , A. Tjønneland , U. Vogel , and T. I. Kopp , “Intake of Red and Processed Meat, Use of Non‐Steroid Anti‐Inflammatory Drugs, Genetic Variants and Risk of Colorectal Cancer: A Prospective Study of the Danish “Diet, Cancer and Health” Cohort,” International Journal of Molecular Sciences 20, no. 5 (2019): 1121. 10.3390/ijms20051121 PMC6429260 30841568

[29] A. Buonacera , B. Stancanelli , M. Colaci , and L. Malatino , “Neutrophil to Lymphocyte Ratio: An Emerging Marker of the Relationships between the Immune System and Diseases,” International Journal of Molecular Sciences 23, no. 7 (2022): 3636. 10.3390/ijms23073636 PMC8998851 35408994

[30] S. Balta , S. Demirkol , M. Unlu , Z. Arslan , and T. Celik , “Neutrophil to lymphocyte ratio may be predict of mortality in all conditions,” British Journal of Cancer 109, no. 12 (2013): 3125–3126. 10.1038/bjc.2013.598 PMC3859933 24084765

[31] J. A. Olzmann and P. Carvalho , “Dynamics and functions of lipid droplets,” Nature Reviews Molecular Cell Biology 20, no. 3 (2019): 137–155. 10.1038/s41580-018-0085-z PMC6746329 30523332

[32] P. Yang , H. Qin , Y. Li , et al., “CD36‐mediated metabolic crosstalk between tumor cells and macrophages affects liver metastasis,” Nature Communications 13, no. 1 (2022): 5782. 10.1038/s41467-022-33349-y PMC9527239 36184646

[33] W. Cui , A. Sathyanarayan , M. Lopresti , M. Aghajan , C. Chen , and D. G. Mashek , “Lipophagy‐derived fatty acids undergo extracellular efflux via lysosomal exocytosis,” Autophagy 17, no. 3 (2021): 690–705. 10.1080/15548627.2020.1728097 PMC8032247 32070194

[34] J. Cerny , Y. Feng , A. Yu , et al., “The small chemical vacuolin‐1 inhibits Ca^2+^‐dependent lysosomal exocytosis but not cell resealing,” The EMBO Reports 5, no. 9 (2004): 883–888. 10.1038/sj.embor.7400243 PMC1299144 15332114

[35] M. De Martino , J. C. Rathmell , and L. Galluzzi , “Vanpouille‐Box C. Cancer cell metabolism and antitumour immunity,” Nature Reviews Immunology 24, no. 9 (2024): 654–669. 10.1038/s41577-024-01026-4 PMC11365797 38649722

[36] H. G. Rodrigues , R. Curi , and M. A. R. Vinolo , “Fatty acids as modulators of neutrophil recruitment, function and survival,” European Journal of Pharmacology 785 (2016): 50–58. 10.1016/j.ejphar.2015.03.098 25987417

[37] S. J. Collins , F. W. Ruscetti , R. E. Gallagher , and R. C. Gallo , “Terminal differentiation of human promyelocytic leukemia cells induced by dimethyl sulfoxide and other polar compounds,” Proceedings of the National Academy of Sciences USA 75, no. 5 (1978): 2458–2462. 10.1073/pnas.75.5.2458 PMC392573 276884

[38] H. Wang , L. Yang , M. Liu , and J. Luo , “Protein post‐translational modifications in the regulation of cancer hallmarks,” Cancer Gene Therapy 30, no. 4 (2023): 529–547. 10.1038/s41417-022-00464-3 35393571

[39] C. Pan , B. Li , and M. C. Simon , “Moonlighting functions of metabolic enzymes and metabolites in cancer,” Molecular Cell 81, no. 18 (2021): 3760–3774. 10.1016/j.molcel.2021.08.031 34547237

[40] M. Qu , X. Zhou , X. Wang , and H. Li , “Lipid‐induced S‐palmitoylation as a Vital Regulator of Cell Signaling and Disease Development,” International Journal of Biological Sciences 17, no. 15 (2021): 4223–4237. 10.7150/ijbs.64046 PMC8579454 34803494

[41] A. Banerjee , “In vitro reconstitution of substrate S‐acylation by the zDHHC family of protein acyltransferases,” Open Biology 12, no. 4 (2022): 210390. 10.1098/rsob.210390 PMC9006032 35414257

[42] C. Cheng , F. Geng , X. Cheng , and D. Guo , “Lipid metabolism reprogramming and its potential targets in cancer,” Cancer Communications 38, no. 1 (2018): 27. 10.1186/s40880-018-0301-4 PMC5993136 29784041

[43] J. Zhu and C. B. Thompson , “Metabolic regulation of cell growth and proliferation,” Nature Reviews Molecular Cell Biology 20, no. 7 (2019): 436–450. 10.1038/s41580-019-0123-5 PMC6592760 30976106

[44] Q. Gu , Y. Wang , P. Yi , and C. Cheng , “Theoretical framework and emerging challenges of lipid metabolism in cancer,” Seminars in Cancer Biology 108 (2025): 48–70. 10.1016/j.semcancer.2024.12.002 39674303

[45] E. Currie , A. Schulze , R. Zechner , T. C. Walther , and R. V. Farese , “Cellular fatty acid metabolism and cancer,” Cell Metabolism 18, no. 2 (2013): 153–161. 10.1016/j.cmet.2013.05.017 PMC3742569 23791484

[46] S. M. Woo , H. O. Lee , J. H. Kang , et al., “Loss of SLC25A20 in Pancreatic Adenocarcinoma Reversed the Tumor‐Promoting Effects of a High‐Fat Diet,” Theranostics 15, no. 13 (2025): 6516–6533. 10.7150/thno.114912 PMC12160018 40521210

[47] X. Cheng , F. Geng , M. Pan , et al., “Targeting DGAT1 Ameliorates Glioblastoma by Increasing Fat Catabolism and Oxidative Stress,” Cell Metabolism 32, no. 2 (2020): 229–242.e8. 10.1016/j.cmet.2020.06.002 PMC7415721 32559414

[48] I. Martínez‐Reyes and N. S. Chandel , “Cancer metabolism: Looking forward,” Nature Reviews Cancer 21, no. 10 (2021): 669–680. 10.1038/s41568-021-00378-6 34272515

[49] A. E. Baek , Y.‐R. A. Yu , S. He , et al., “The cholesterol metabolite 27 hydroxycholesterol facilitates breast cancer metastasis through its actions on immune cells,” Nature Communications 8, no. 1 (2017): 864. 10.1038/s41467-017-00910-z PMC5636879 29021522

[50] C. Rosales , “Neutrophil: A Cell with Many Roles in Inflammation or Several Cell Types?,” Frontiers in Physiology 9 (2018): 113. 10.3389/fphys.2018.00113 PMC5826082 29515456

[51] C. Salvagno , M. Ciampricotti , S. Tuit , et al., “Therapeutic targeting of macrophages enhances chemotherapy efficacy by unleashing type I interferon response,” Nature Cell Biology 21, no. 4 (2019): 511–521. 10.1038/s41556-019-0298-1 PMC6451630 30886344

[52] M. Zhang , L. Zhou , Y. Xu , et al., “A STAT3 palmitoylation cycle promotes TH17 differentiation and colitis,” Nature 586, no. 7829 (2020): 434–439. 10.1038/s41586-020-2799-2 PMC7874492 33029007

[53] T. Das , J. S. Yount , and H. C. Hang , “Protein S‐palmitoylation in immunity,” Open Biology 11, no. 3 (2021): 200411. 10.1098/rsob.200411 PMC8061762 33653086

[54] S. Lang , K. Bruderek , C. Kaspar , et al., “Clinical Relevance and Suppressive Capacity of Human Myeloid‐Derived Suppressor Cell Subsets,” Clinical Cancer Research 24, no. 19 (2018): 4834–4844. 10.1158/1078-0432.CCR-17-3726 29914893

[55] M. Li , Y. Yang , L. Xiong , P. Jiang , J. Wang , and C. Li , “Metabolism, metabolites, and macrophages in cancer,” Journal of Hematology & Oncology 16, no. 1 (2023): 80. 10.1186/s13045-023-01478-6 PMC10367370 37491279

[56] T. M. Grzywa , A. Sosnowska , P. Matryba , et al., “Myeloid Cell‐Derived Arginase in Cancer Immune Response,” Frontiers in Immunology 11 (2020): 938. 10.3389/fimmu.2020.00938 PMC7242730 32499785

[57] Z. Erpapazoglou , O. Walker , and R. Haguenauer‐Tsapis , “Versatile roles of k63‐linked ubiquitin chains in trafficking,” Cells 3, no. 4 (2014): 1027–1088. 10.3390/cells3041027 PMC4276913 25396681

[58] F. SM , L. Abrami , M. E. Linder , S. X. Bamji , B. C. Dickinson , and F. G. van der Goot , “Mechanisms and functions of protein S‐acylation,” Nature Reviews Molecular Cell Biology 25, no. 6 (2024): 488–509. 10.1038/s41580-024-00700-8 PMC12010433 38355760

[59] S. J. Kridel , F. Axelrod , N. Rozenkrantz , and J. W. Smith , “Orlistat is a novel inhibitor of fatty acid synthase with antitumor activity,” Cancer Research 64, no. 6 (2004): 2070–2075. 10.1158/0008-5472.can-03-3645 15026345

[60] M. Wilhelm , L. Rossmeislová , and M. Šiklová , “Approaches to Measuring the Activity of Major Lipolytic and Lipogenic Enzymes In Vitro and Ex Vivo,” International Journal of Molecular Sciences 23, no. 19 (2022): 11093. 10.3390/ijms231911093 PMC9570359 36232405

[61] D. V. F. Tauriello , S. Palomo‐Ponce , D. Stork , et al., “TGFβ drives immune evasion in genetically reconstituted colon cancer metastasis,” Nature 554, no. 7693 (2018): 538–543. 10.1038/nature25492 29443964

[62] S.‐S. Hu , Y. Han , T.‐Y. Tan , et al., “SLC25A21 downregulation promotes KRAS‐mutant colorectal cancer progression by increasing glutamine anaplerosis,” JCI Insight 8, no. 21 (2023): 167874. 10.1172/jci.insight.167874 PMC10721270 37937641

[63] A. Ali , E. Levantini , J. T. Teo , et al., “Fatty acid synthase mediates EGFR palmitoylation in EGFR mutated non‐small cell lung cancer,” EMBO Molecular Medicine 10, no. 3 (2018): e8313. 10.15252/emmm.201708313 PMC5840543 29449326

[64] S. C. Kwong , A. H. A. Jamil , A. Rhodes , N. A. Taib , and I. Chung , “Metabolic role of fatty acid binding protein 7 in mediating triple‐negative breast cancer cell death via PPAR‐α signaling,” Journal of Lipid Research 60, no. 11 (2019): 1807–1817. 10.1194/jlr.M092379 PMC6824484 31484694

[65] S. A. Lagerstedt , D. R. Hinrichs , S. M. Batt , M. J. Magera , P. Rinaldo , and J. P. McConnell , “Quantitative Determination of Plasma C8–C26 Total Fatty Acids for the Biochemical Diagnosis of Nutritional and Metabolic Disorders,” Molecular Genetics and Metabolism 73, no. 1 (2001): 38–45. 10.1006/mgme.2001.3170 11350181

