# Supplementary material for: Epithelial SLC25A20 Deficiency Drives Immunosuppressive Neutrophil Remodeling via ARG1 Palmitoylation to Promote Colorectal Cancer
Source: Adv Sci (Weinh). 2026 Oct 4:e78114. Online ahead of print. doi: 10.1002/advs.78114 (PMC13634579; doi:10.1002/advs.78114)
Supplement: Supplementary file 1 [file ADVS-9999-e78114-s001.docx]

**Title：**

**Epithelial SLC25A20 Deficiency Drives Immunosuppressive Neutrophil Remodeling via ARG1 Palmitoylation to Promote Colorectal Cancer**

Yue Han^1*^, Tian-Yuan Tan^1,2*^, Bin Zhang^1,2^, Ye-Bin Fu^1^, Wen Chen^1^, Min-Hui Yang^1^, Yi-Qing Wang^1^, Yan-Qing Ding^1,3#^, Shuang Wang^1,2,3#^

**Supplementary Methods**

**Cell culture**

The human CRC cell lines RKO, Caco-2 (RRID: CVCL_0025), LS174T (RRID: CVCL_1384), SW620, HCT116 (RRID: CVCL_0291), HT29 (RRID: CVCL_0320), SW480 (RRID: CVCL_0546), HCT8, LoVo (RRID: CVCL_0399), DLD1, and a normal colon mucosal epithelial cell line NCM460 (RRID: CVCL_0460) were gained from ATCC. M5 cells are liver derived subclones obtained by our laboratory through in vivo screening and isolation of SW480 cells, with strong metastatic ability. Human promyelocytic leukemia HL-60 (RRID: CVCL_0002) cells and mouse colon cancer MC38 (RRID: CVCL_B288) cell lines were purchased by Guangzhou Taylor Biotechnology Co., Ltd. All cell lines were authenticated by short tandem repeat profiling, and all lines routinely screened for Mycoplasma and were identified to be negative throughout the study. All CRC cell lines and NCM460 cells were cultured in RPMI-1640 medium (Invitrogen) containing 10% fetal bovine serum (FBS) (Invitrogen) at 37 °C in a cell incubator containing 5% CO_2_. MC38 cells and HL-60 cells were cultured using DMEM (Invitrogen) and IMDM (Invitrogen) containing 10% FBS, respectively, under the same conditions as CRC cells.

**Western blotting analysis**

Total protein was extracted from tissues and cells by RIPA lysis solution mixed with phosphatase inhibitors, protease inhibitors, and PMSF. Proteins were separated by 10% SDS-PAGE and transferred onto polyvinylidene fluoride (PVDF) membranes (Millipore, Darmstadt, Germany, catalog IPVH00010). PageRuler Prestained Protein Ladder (ThermoFisher Scientific, catalog 26616) was used as the molecular weight marker. Subsequently, membranes were blocked with 5% BSA solution prepared in 1 x PBS-T (0.5% Tween-20) for 1 h. Membranes were incubated with the target primary antibody at 4 °C overnight. HRP-labeled secondary antibodies were incubated at room temperature for 1 h. Blots were visualized with ECL western blotting Substrate (ThermoFisher Scientific, IL, USA). All antibodies used for western blotting are listed in Supplementary Table S5.

**TCGA analysis**

The TCGA dataset was applied to identify the expression levels of SLC25A20 in 622 human CRC tissues and 51 normal tissues.

**Flow cytometry detection**

Tumor tissues from each group of mice were separated and cut into pieces, with adding tissue digestion solution containing 0.5 mg/ml collagenase IV and 0.1 mg/ml DNase I. The whole system was digested at 37 °C for 30 min. Then 70 μm pore size filter was used to filter the above suspension, after centrifuging at 350 g for 5 min at 4 °C and adding cold red blood cell lysis solution (Solarbio, Beijing, China). The samples were washed and resuspend with PBS. Filtering again through the 40 μm cell filter and centrifuging to obtain Peripheral blood mononuclear cells (PBMCs). The anti Cd45 (RRID: AB_312972), Cd3 (RRID: AB_1595597), Cd4 (RRID: AB_493698), Cd8 (RRID: AB_312760), Cd25 (RRID: AB_312860), Cd19 (RRID: AB_2572119), F4/80 (RRID: AB_10901171), Cd11b (RRID: AB_2561390), and Ly6g (RRID: AB_1186104) of mice were reacted under dark conditions for 1 h, and the proportion of various immune cells was analyzed by flow cytometry. All antibodies used for flow cytometry detection are listed in Supplementary Table S5.

**In vivo tumorigenic and metastasis assays**

All animal experiments were performed in strict accordance with the recommendations in the Guide for the Care and Use of Laboratory Animals of the National Institutes of Health. The protocol was approved by the Animal Ethics Committee of Nanfang Hospital, Southern Medical University (No. IACUC-LAC-20250811-001). Balb/C-nu/nu nude mice (4-5 weeks old) and C57BL/6 mice (RRID: IMSR_JAX:000664; 4-5 weeks old) were obtained from the Laboratory Animal Center of Nanfang Hospital, Southern Medical University. A specific-pathogen-free condition and an autoclaved laboratory rodent diet were provided to mice. Before the experiment, mice were randomly divided into groups according to the principle of randomization. Given that sex as a biological variable, we selected mice of the same gender for experiments.

To evaluate in vivo tumorigenicity, 5 × 10^6^ RKO cells stably with SLC25A20 knockout and the control cells were subcutaneously injected into the dorsal flanks of 4-week-old BALB/c (nu/nu) nude mice (RRID: MMRRC_000103-UCD), respectively. Using the same method to subcutaneously inoculate SLC25A20 knockout and control MC38 cells into 4-week-old C57BL/6 mice. Tumor volume was recorded every three days and calculated according to the following formula: V =0.5 × length × wildth^2^. After 18-21 days, the mice were euthanized under anesthesia and the tumors were separated for subsequent analysis.

For neutrophil depletion in tumor-bearing mice, once subcutaneous tumors reached 100-120mm^3^ (day 7 post-tumor inoculation), the anti-Ly6G antibody (Biolegend,127649) at a dose of 25 mg/kg or PBS were delivered by tail-vein injection every day until the experimental endpoint. To verify neutrophil depletion efficiency, tumor tissues after red blood cell lysis using lysis buffer, the frequency of Ly6G^+^Mpo^+^neutrophils was quantified via flow cytometry and IHC was used to detect Mpo.

For pharmacological inhibition of palmitoylation or FASN in tumor-bearing mice, once subcutaneous tumors reached 100-120 mm^3^ (day 7 post-tumor inoculation), 2-BP (Sigma-Aldrich, 238422-10G, 40 mg/kg) or Orlistat (KKL Med, KM13746, 50 mg/kg) was administered intraperitoneally every day until experimental endpoint. As for PA (Sigma-Aldrich, 57-10-3, 10 mg/kg) was administered by oral gavage. At experimental endpoint, tumor tissues were harvested.

For immune checkpoint blockade therapy, treatment was initiated when subcutaneous tumors reached 100-120 mm^3^ (day 7 post-tumor inoculation). Anti-mouse PD-1 antibody (Selleck, A2122) diluted in sterile PBS was administered intraperitoneally at 10 mg/kg per mouse every 4 days until experimental endpoint.

The maximum diameter of tumors all less than 15  mm which meeting the approved animal protocol.

The in vivo metastasis model was established to assess lung metastasis. 5-week-old BALB/c (nu/nu) nude mice were injected intravenously via the lateral tail vein with 2 x 10^6^ RKO cells. The same method was applied to inject MC38 cells into 5-week-old C57BL/6 mice. After 4 weeks, the mice were euthanized after anesthesia. The lungs were separated for fixing with 10% neutral-buffered formalin. Eventually, the metastatic nodules of the lungs were observed and counted under the microscope after hematoxylin-eosin (H&E) staining.

To establish the hepatic metastasis model, 6-8-week-old C57BL/6 mice were anesthetized with isoflurane. A small left subcostal laparotomy was performed, and the spleen was gently exteriorized. MC38 colorectal cancer cells (5 × 10⁵ SLC25A20 knockout and the control cells in 80 μL sterile PBS) were slowly injected beneath the splenic capsule using a needle. The needle was kept in place for 90s before slow withdrawal, and the injection site was compressed with moist sterile cotton swabs to prevent bleeding and tumor cell leakage. After injection, splenectomy was performed following ligation of the splenic vessels to eliminate primary splenic tumor burden. The abdominal wall and skin were closed with absorbable sutures. Mice were kept on a warming pad for recovery and received postoperative analgesia. Mice were sacrificed 21-24 days after surgery. Livers were harvested for macroscopic metastasis quantification and histological staining.

To establish the cecal orthotopic colorectal cancer model, 6-8-week-old C57BL/6 mice were anesthetized with isoflurane. A small mid-abdominal laparotomy was performed, and the cecum was gently exteriorized and kept moist with warm sterile saline. MC38 cells (1 × 10⁶ cells mixed 1:1 with ice-cold Matrigel in a total volume of 50 μL) were injected into the sub-serosal layer of the cecal wall using a needle. The needle was held in place for 60-90s before slow withdrawal, and the injection site was compressed with moist cotton swabs to prevent leakage of tumor cells. The cecum was carefully returned to the peritoneal cavity. The abdominal muscle and skin were closed with absorbable sutures. Mice were recovered on a warming pad and received postoperative analgesia. Mice were sacrificed 21-28 days after surgery. Primary cecal tumors were harvested for flow cytometry analysis.

**Analysis of FFA content**

The content of FFA was detected according the ELISA kit (Abmart, Shanghai, China). A total of 50 µL cell-free supernatant from different treatment cells was added to a coated well plate with specific antibodies at 37 °C for 1 h incubation. After washing 5 times, then 100 µL Enzyme-linked reagent was added and with another incubation at 37 °C for 30 min. After 5 times washing repeated, each well reacted in dark at 37 °C for 15 min after adding A and B (50 µL) and then 50 µL stop solution to end reaction. The absorbance value was measured at a wavelength of 450 nm and the standard curve was plotted to calculate the FFA content of each sample.

**Lipid droplet dyeing and quantification with Bodipy**

Lipid droplets in living cells were stained with BODIPY 493/503 (0.5 μM) for 30 min and observed under the Olympus Fluo View FV1000 confocal laser scanning microscope. The nucleus therein was stained with DAPI (Solarbio, Beijing, China).

**Induction of dHL-60 cell differentiation**

Human promyelocytic leukemia HL-60 cells were cultured in IMDM (Invitrogen, C12440500BT) containing 10% FBS and 1.25% DMSO in 5% CO_2_ at 37 °C for 8 days to obtain neutrophil-like cells (dHL-60).

**Cytological staining**

The differentiated dHL-60 cells were collected by centrifugation and processed into cell wax after fixation, dehydration, transparency, waxing and embedding. The morphological changes of the nucleus were observed under an optical microscope after slicing and staining.

**Establishment of the co-culture system for CRC cells and immune cells**

Transwell with a 0.4 μm pore membrane were installed into the matching 6-well plates, and a total of 2 × 10^5^ human CRC cells with different SLC25A20 expression or treated with different methods, as well as their respective control cells, were inoculated into the lower chamber. Then, dHL-60 cells that have been induced completely were added to the upper chamber according to 5 times the number of tumor cells in order to establish a tumor cell-neutrophil co culture system in vitro. The system was continuously cultured for 72 h for subsequent experimental research. When observing the effect of CD8^+^T cells, a mixture of dHL-60 cells and CD8^+^T cells was seeded into the upper chamber of transwell.

**Seahorse Analysis**

The Seahorse XFe 24 Extracellular Flux Bioanalyzer (Agilent) was used to measure oxygen consumption rate (OCR) and extracellular acidification rate (ECAR) according to the manufacturer’s protocol. Cells were placed into fresh DMEM medium containing 10 mM glucose, 2 mM L-glutamine, and 1 mM sodium pyruvate and incubated for 1 h. Then, three metabolic inhibitors were sequentially loaded into each well, oligomycin (Oligo, 1 μM), followed by carbonyl cyanide 4-trifluoromethoxyphenylhydrazone (FCCP, 2 μM), followed by rotenone (Rot, 2 μM).

**Click-chemistry-based detection of exogenously expressed ARG1 palmitoylation**

To specifically assess palmitoylation of exogenously expressed ARG1 in dHL-60 cells, parental HL-60 cells were transfected with FLAG-tagged wild-type ARG1 (FLAG-ARG1-WT), palmitoylation-deficient mutant FLAG-ARG1-C303A, or empty FLAG vector. After transfection, cells were differentiated into dHL-60 cells with 1.25% DMSO for 8 days. For metabolic labeling of palmitoylated proteins, differentiated dHL-60 cells were cultured in RPMI-1640 medium supplemented with 2% charcoal-dextran-stripped FBS and treated with 50 μM Click-iT palmitic acid azide (C10265) or equal volume of DMSO for 16-18 h. Cells were lysed in RIPA buffer containing protease inhibitors and 50 mM N-ethylmaleimide (NEM) to block free thiol groups. Exogenous FLAG-tagged ARG1 was immunoprecipitated with anti-FLAG M2 affinity gel. Bead-bound proteins were subjected to copper-catalyzed azide‑alkyne cycloaddition (CuAAC) click-chemistry reaction with alkyne-biotin probe. EDTA was added to quench the reaction. After extensive washing, proteins were eluted and analyzed by Western blotting. Palmitoylation signals were detected by streptavidin-HRP, and total immunoprecipitated ARG1 was validated using anti-FLAG antibody.

**Chemicals**

Small-molecular compounds such as Etomoxir (Selleck, S8244), 2-BP (Sigma-Aldrich, 238422-10G), Cycloheximide (Sigma-Aldrich, C1988), Orlistat (KKL Med, KM13746), Chloroquine (KKL Med, KM17589), 3-Methyladenine (KKL Med, KM17736), MG-132 (KKL Med, KM2291), DEVD-CHO (KKL Med, KM15411).

**Supplementary Figures S1-S18**

Figure S1. SLC25A20 downregulation is associated with CRC malignant progression. (A) GSEA enrichment analysis to observe the relationship between SLC25A20 expression and tumor invasion. (B) GSEA enrichment analysis to observe the relationship between SLC25A20 expression and tumor metastasis. (C) Quantitative analysis of SLC25A20 protein expression in 24 paired CRC and adjacent non-tumor tissues. Protein levels were determined by immunoblotting, and statistical analysis was performed using a paired two-sided t-test. Data are presented as mean ± SD. Statistical significance was calculated by paired two-sided t test (C) ; the *P* values are shown.

Figure S2. The expression levels of SLC25A20 in CRC cells. (A) Real-time PCR for SLC25A20 mRNA detection in human CRC cells with or without SLC25A20 overexpression or knockout. (B) Immunoblot analysis of SLC25A20 protein in human CRC cells with or without SLC25A20 overexpression or knockout. (C) Real-time PCR for SLC25A20 mRNA detection in mouse MC38 cells with or without SLC25A20 knockout. (D) Immunoblot analysis of SLC25A20 protein in mouse MC38 cells with or without SLC25A20 knockout. Original uncropped and unaltered blots for this panel are provided in Supplementary Figure S17. Data are presented as the mean ± SD. Statistical significance was calculated unpaired 2-sided t test (A and C). **P* < 0.05, ***P* < 0.01, ****P* < 0.001, and *****P* < 0.0001.

Figure S3. Enhanced anti-tumor activity of SLC25A20 in immunocompetent mice and validation of neutrophil depletion efficiency in vivo. (A) Schematic diagrams illustrating the establishment of xenograft models in nude mice. (B) Quantification of tumor weights derived from CRC cell xenografts in nude mice. (C) Schematic diagrams illustrating the establishment of xenograft models in C57BL/6 mice. (D) Quantification of tumor weights derived from CRC cell xenografts in C57BL/6 mice. (E) IHC staining verifying the in-vivo neutrophil-depletion efficiency. Data are represented as the mean ± SD. Statistical significance was calculated unpaired 2-sided t test (B and D). **P* < 0.05; ***P* < 0.01; ****P* < 0.001; *****P* < 0.0001.

Figure S4. The detection of intracellular lipid levels in CRC cells. (A) Lipidomic analysis for detecting FA levels in SLC25A20 KO cells (n=6). (B) Representative images of lipid droplets stained with Oil red O (left) and quantification (right) for SLC25A20 knockout and control CRC cells. (C) Relative transcript levels of CD36 in CRC cells with or without SLC25A20 knockout. (D) Relative transcript levels of CD36 in CRC cells with or without CD36-siRNA transfection by Real-time PCR. (E) The MFI of Bodipy in CD36 knockdown and control CRC cells was measured by flow cytometry. (F) Relative transcript levels of ATGL and DGAT1/2 in CRC cells with or without their respective siRNA transfection by Real-time PCR. (G) The FFA levels in SLC25A20 knockout and control RKO cells treated with ATGL-siRNA or DGAT1/2-siRNA Experiments (B)-(G) were repeated at least three times and representative data are shown. Data are represented as the mean ± SD. Statistical significance was calculated unpaired 2-sided t test (B-D, E-F ) and one-way ANOVA with Tukey’s post hoc test (G). **P* < 0.05; ***P* < 0.01; ****P* < 0.001; *****P* < 0.0001.

Figure S5. Identification of dHL-60 cell differentiation and interference effects. (A) CD11b protein expression during dHL-60 differentiation induced by DMSO was measured on hours 24, 48, 96 and 144 using western blot. (B) Relative transcript levels of *CD36* in dHL-60 cells with or without CD36-siRNA infection. Original uncropped and unaltered blots for this panel are provided in Supplementary Figure S17. Data are presented as the mean ± SD. Statistical significance was calculated unpaired 2-sided t test (B). **P* < 0.05, ***P* < 0.01, ****P* < 0.001, and *****P* < 0.0001.

Figure S6. Quantification of representative chemokines and TGF-β in culture supernatants from SLC25A20-knockout and control CRC cells (n=5). Data are presented as the mean ± SD. Statistical significance was calculated unpaired 2-sided t test. **P* < 0.05, ***P* < 0.01, ****P* < 0.001, and *****P* < 0.0001.

Figure S7. Lipid-driven alterations of ARG1 protein stability in neutrophils triggered by SLC25A20-deficient CRC cells. (A) Lipidomics analysis of differential lipid metabolites in supernatant from SLC25A20 knockout or control RKO cells (n = 6 per group). (B) Dose-response assays of dHL-60 cells treated with gradient concentrations of exogenous palmitic acid. (C) Quantification of ARG1 protein levels from CHX-chase assays. dHL-60 cells were treated with or without exogenous palmitic acid, and whole-cell lysates were harvested at indicated time points. ARG1 turnover was assessed by immunoblotting. Original uncropped and unaltered blots for this panel are provided in Supplementary Figure S17.

Figure S8. The construction of palmitoylation-deficient mutant C303A. (A) The Palmitoylation Sites of ARG1 predicted by CSS palm 4.0. (B) The Palmitoylation Sites of ARG1 searched from the SwissPalm S-acylation database. (C and D) ARG1 vector schematic diagram (C) and sequencing results (D) after mutation.

Figure S9. The expression levels of ZDHHC12 and ZDHHC18. (A) 23 human palmitoyltransferase ZDHHCs expression levels in neutrophils analyzed through The Human Protein Atlas. (B) IP-MS-based identification of ZDHHC18 as an ARG1-interacting partner. (C) Relative transcript levels of ZDHHC12 and ZDHHC18 in dHL-60 cells with/without ZDHHC12-siRNA or ZDHHC18-siRNA treatment (n = 3). (D) The interference efficiency of ZDHHC12-siRNA and ZDHHC18-siRNA in macrophages was confirmed by Real-time PCR (n = 3). Data are presented as the mean ± SD. Statistical significance was calculated unpaired 2-sided t test (C-D). **P* < 0.05, ***P* < 0.01, ****P* < 0.001, and *****P* < 0.0001.

Figure S10. Identification of the palmitoyltransferase in ARG1. (A) The ARG1 transcript levels in dHL-60 cells transfected with control or ZDHHC12/18 siRNA for 48 h were quantified by real-time PCR (n = 3 biologically independent experiments). (B) Immunoblot analysis of ARG1 protein in THP-1-derived macrophages with or without ZDHHC12 and ZDHHC18 siRNA transfection. Anti-tubulin was used as a loading control. (C) The levels of palmitoylated ARG1 in THP-1-derived macrophages with ZDHHC12 or ZDHHC18 knockdown were visualized by ABE assay and immunoblot analysis. (D) The interaction between ARG1 and ZDHHC18 in THP-1-derived macrophages was revealed by Co-IP and Immunoblot analysis. (E) THP-1-derived macrophages were incubated with or without palmitic acid or 2BP for 48h, followed by Co-IP and immunoblotting. Original uncropped and unaltered blots for this panel are provided in Supplementary Figure S18. Data are presented as the mean ± SD. Statistical significance was calculated one-way ANOVA with Dunnett’s post hoc test (A); the P values are shown.

Figure S11. The palmitoylation level of neutrophils affects their immunosuppressive functions. (A-B) Representative micrographs (A) and quantification (B) for displaying the colony-forming ability of of CRC cells from dHL-60-CD8^+^ T-CRC cell co-culture system (n = 3). Statistical significance was calculated by and one-way ANOVA with Dunnett’s post hoc test (B).****P* < 0.001.

Figure S12.Immunophenotypic alterations of TANs and CD8^+^ T-cells upon 2-BP or Orlistat treatment in vivo. (A) Representative fluorescence imaging staining of SLC25A20 (yellow), LY6G (red), CD8 (white) and nuclear (DAPI, blue) in subcutaneous tumor tissues of C57BL/6 mice treated with SLC25A20 KO in combination or without 2-BP or Orlistat. (B and C) Flow cytometry-based analysis of specific molecule expression on TANs and CD8^+^ T cells infiltrating subcutaneous tumors (B) or cecal orthotopic tumors (C). ns denotes nonsignificant. Data are presented as the mean ± SD. Statistical significance was calculated by one-way ANOVA with Tukey’s post hoc test (B-C). **P* < 0.05, ***P* < 0.01, ****P* < 0.001, and *****P* < 0.0001.

Figure S13.Immunophenotypic alterations of TANs and CD8^+^ T-cells upon an anti-PD-1 mAb, an SLC25A20 overexpression treatment or the anti-PD-1 mAb combined with SLC25A20 overexpression treatment in vivo. (A) Representative fluorescence imaging staining of SLC25A20 (yellow), LY6G (red), CD8 (white) and nuclear (DAPI, blue) (n = 5) in subcutaneous tumor from MC38 tumor-bearing mice treated with an isotype control, an anti-PD-1 mAb, an SLC25A20 overexpression treatment or the anti-PD-1 mAb combined with SLC25A20 overexpression treatment. (B and C) Flow cytometry-based analysis of specific molecule expression on TANs and CD8^+^ T cells infiltrating subcutaneous tumors (B) or cecal orthotopic tumors (C). ns denotes nonsignificant. Data are presented as the mean ± SD. Statistical significance was calculated by one-way ANOVA with Dunnett’s post hoc test (B-C). **P* < 0.05, ***P* < 0.01, ****P* < 0.001, and *****P* < 0.0001.

**B**

**A**

SLC25A20

25

35

KDa

55

70

25

35

55

70

KDa

SLC25A20

Tubulin

Tubulin

Figure S14. Uncropped and unaltered original western blot images for the western blot panels presented in Figure 1 of the main manuscript. (A) Full blots corresponding to Figure 1C. (B) Full blots corresponding to Figure 1E. No digital modifications were performed on these blot images.

**A**

40

KDa

35

ARG1

55

70

Tubulin

55

70

**B**

40

KDa

35

ARG1

Tubulin

40

35

KDa

ARG1

55

70

Tubulin

40

55

70

**C**

KDa

35

ARG1

Tubulin

40

KDa

35

ARG1

55

70

Tubulin

40

55

70

**G**

KDa

35

ARG1

Tubulin

**H**

40

KDa

35

ARG1

55

70

Tubulin

**F**

40

KDa

35

input ARG1

40

KDa

35

Click-iT pulldown

ARG1

40

55

70

**D**

KDa

40

**E**

KDa

40

35

35

35

ARG1

Tubulin

ARG1_palm_

ARG1

Figure S15. Uncropped and unaltered original western blot images for the western blot panels presented in Figure 5 of the main manuscript. (A) Full blots corresponding to Figure 5B. (B) Full blots corresponding to Figure 5D and the right portion of Figure 5F. (C) Full blots corresponding to Figure 5E. (D) Full blots corresponding to the left portion of Figure 5F. (E) Full blots corresponding to Figure 5G. (F) Full blots corresponding to Figure 5H. (G) Full blots corresponding to Figure 5I. (H) Full blots corresponding to Figure 5J. No digital modifications were performed on these blot images.

40

KDa

35

input ARG1

55

70

Tubulin

40

KDa

35

Click-iT pulldown

ARG1

**C**

40

55

70

**A**

KDa

40

35

35

ARG1

Tubulin

Flag-ARG1

40

**B**

KDa

40

35

35

ARG1_palm_

ARG1

**D**

KDa

55

70

40

35

ARG1

Tubulin

**E**

55

70

55

70

40

35

KDa

40

35

ARG1

Tubulin

ARG1

Tubulin

**F**

KDa

55

70

40

KDa

40

55

70

35

35

KDa

40

70

55

35

ARG1

Tubulin

ARG1

Tubulin

40

**J**

KDa

55

40

35

**I**

KDa

55

70

40

35

**K**

KDa

55

40

55

40

40

35

40

35

40

35

40

35

55

40

55

40

**G**

KDa

55

70

40

35

40

**H**

KDa

40

35

35

ARG1

Tubulin

ARG1_palm_

ARG1

ARG1

Tubulin

ARG1

ZDHHC18

ZDHHC18

ARG1

ZDHHC18

ARG1

ARG1

ZDHHC18

ARG1

ZDHHC18

Figure S16. Uncropped and unaltered original western blot images for the western blot panels presented in Figure 6 of the main manuscript. (A) Full blots corresponding to Figure 6A. (B) Full blots corresponding to Figure 6B. (C) Full blots corresponding to Figure 6C. (D) Full blots corresponding to Figure 6D. (E) Full blots corresponding to Figure 6E. (F) Full blots corresponding to Figure 6F. (G) Full blots corresponding to Figure 6G. (H) Full blots corresponding to Figure 6H. (I) Full blots corresponding to Figure 6I. (J) Full blots corresponding to Figure 6K. (K) Full blots corresponding to Figure 6L. No digital modifications were performed on these blot images.

**A**

55

70

25

35

KDa

55

70

25

35

**B**

KDa

25

35

55

70

SLC25A20

Tubulin

SLC25A20

Tubulin

SLC25A20

Tubulin

**C**

130

170

55

70

KDa

CD11b

Tubulin

40

35

ARG1

55

70

Tubulin

**D**

Figure S17. Uncropped and unaltered original western blot images for the western blot panels presented in Supplementary Figures 2, 5, and 7. (A and B) Full blots corresponding to Supplementary Figure 2B (A) and Supplementary Figure 2D (B). (C) Full blots corresponding to Supplementary Figure 5A. (D) Full blots corresponding to Supplementary Figure 7B. No digital modifications were performed on these blot images.

**A**

KDa

55

70

40

35

40

**B**

KDa

40

35

35

40

**C**

KDa

55

40

35

**D**

KDa

55

40

40

35

55

40

40

35

40

35

40

35

55

40

55

40

ARG1

Tubulin

ARG1_palm_

ARG1

ARG1

ZDHHC18

ZDHHC18

ARG1

ZDHHC18

ARG1

ARG1

ZDHHC18

ARG1

ZDHHC18

Figure S18. Uncropped and unaltered original western blot images for the western blot panels presented in Supplementary Figures 10. (A) Full blots corresponding to Supplementary Figure 10B. (B) Full blots corresponding to Supplementary Figure 10C. (C) Full blots corresponding to Supplementary Figure 10D. (D) Full blots corresponding to Supplementary Figure 10E. No digital modifications were performed on these blot images.

**Tables S1-S5**

| **Table S1.** The sequences of CRISPR-Cas9 gRNA and the lentivirus primers for SLC25A20 | |
| --- | --- |
| Gene name | Sequence (5’-3’) |
| SLC25A20-gRNA 1 | GGTTGTCCAGGCAAACTCGGTGG |
| SLC25A20-gRNA 2 | AGTCTTCCGGAAACAGTCAAAGG |
| SLC25A20-gRNA 3 | ACACAGCCACCGAGTTTGCCTGG |
| SLC25A20-gRNA 4 | GGACAACCTCCCATGTACTCTGG |
| Lentivirus-5’primer | GAAGTTAGGCCAGCTTGGCACTTG |
| Lentivirus-3’primer | CATGGCGGTCATATTGGAC |

| **Table S2.** Sequences of primers used for qRT-PCR | |
| --- | --- |
| Gene name | Sequence (5’-3’) |
| SLC25A20-F | TCGATTCCAGACTGCACCTC |
| SLC25A20-R | GGGGTGGCCCAATTAAGGAA |
| GAPDH-F | GGACCTGACCTGCCGTCTAG |
| GAPDH-R | GTAGCCCAGGATGCCCTTGA |
| ACSL4-F | ACTGGCCGACCTAAGGGAG |
| ACSL4-R | GCCAAAGGCAAGTAGCCAATA |
| ACSL5-F | CTCAACCCGTCTTACCTCTTCT |
| ACSL5-R | GCAGCAACTTGTTAGGTCATTG |
| CPT1A-F | TGCCCTGAGACGGGGATTAT |
| CPT1A-R | CCAGCCCAGCACATGAACTT |
| CPT1B-F | CCTGCTACATGGCAACTGCTA |
| CPT1B-R | AGAGGTGCCCAATGATGGGA |
| CPT1C-F | GGGCCGCTTTCTTTGTGTC |
| CPT1C-R | AGAAGACGATTAGGGTGAAGGAT |
| CPT2-F | AAGAAGCAGCAATGGGCCAG |
| CPT2-R | AGGGTCCAGGTAGAGCTCAG |
| ACAD10-F | CAAACACTCGGCCTGTGAAAA |
| ACAD10-R | ACTAGATCACGATTAGCCAGCC |
| ACADL-F | CCACAGGTGGACGCCTCA |
| ACADL-R | AATGAGAACATCGCGCGGC |
| HADHB-F | AGCTGTCCAGACCAAAACGA |
| HADHB-R | TGCAACAAACCCGAAGTGC |
| ACAD11-F | TTGGATTCCCCGTTCCCAAG |
| ACAD11-R | AAATCACGGAAGATTCGACCC |
| HADHA-F | ATATGCCGCAATTTTACAGGGT |
| HADHA-R | CAGTGATCTGGAATCACCGCT |
| HADH-F | AAAGAGGTTGGCCTCAGCAG |
| HADH-R | ATCTCCGCAGGGTCATGTTC |
| ECH1-F | ATAGTGGCTTCTCGCAGACTC |
| ECH1-R | CAGTGAGGCGAAGGCTAATAC |
| ACSL1-F | TGTTGACAAGCCAGAGAAG |
| ACSL1-R | CTCGTTCCACCAGTTCAC |
| ACSL3-F | GCCGAGTGGATGATAGCTGC |
| ACSL3-R | ATGGCTGGACCTCCTAGAGTG |
| ACSL6-F | GTGTTGGCTTCTTCCAGGGAGA |
| ACSL6-R | GGTGTGTTTGCCTGGCTGAAGA |
| ATGL-F | CCAACACCAGCATCCAGTTC |
| ATGL-R | ATCCCTGCTTGCACATCTCT |
| DGAT1-F | CCTACCGCGATCTCTACTACTT |
| DGAT1-R | GGGTGAAGAACAGCATCTCAA |
| DGAT2-F | GCTGACCACCAGGAACTATATC |
| DGAT2-R | GGGAACTTCTTGCTCACTTCT |
| ARG1-F | GCAGAAGTCAAGAAGAACGG |
| ARG1-R | GGTTGTCAGTGGAGTGTTG |
| CD36-F | TCCTGCAGAATACCATTTGATCCT |
| CD36-R | TGGTTTCTACAAGCTCTGGTTCTTA |
| ZDHHC12-F | CTGCACGATACCGAGCTGC |
| ZDHHC12-R | CACATTCACGTAGCCAGGGT |
| ZDHHC18-F | CGACAACTGTGTGGAACGAT |
| ZDHHC18-R | AGGAAGTTGCTTCCCTGAGC |
| Slc25a20-F | CATGTGCCTGGTGTTTGTGG |
| Slc25a20-R | CCCTGTGATGCCCTCTCTCA |

| **Table S3.** The sequences of siRNA | |
| --- | --- |
| Gene name | Sequence (5’-3’) |
| DGAT1-siRNA | TGTGCTACGAGCTCAACTTTC |
| DGAT2-siRNA | GTTCTAGGTGGTGGCTAAATC |
| ATGL-siRNA | GCGAGAAGACGTGGAACATCT |
| CD36-siRNA | GACGUUAAUCUGAAAGGAATT |
| ZDHHC18 | GGCAGAUGGUGAAGCUGAAGU |
| ZDHHC12 | CGCUGGUGCUCUUCCUGCACG |
| negative control | UUCUCCGAACGUGUCACGUTT |

| **Table S4.** The sequences of primers used for ARG1 mutant | |
| --- | --- |
| Gene name | Sequence (5’-3’) |
| ARG1-F | CAGGATCCATGAGCGCCAAGTCCAGAAC |
| ARG1-R | CGGCTCGAGTTACTTAGGTGGGTTAAGGTAGTCAATAGG |
| ARG1-MUT-F | AACCTTGGCTGCGTTCGGACTTGCTC |
| ARG1-MUT-R | GAGCAAGTCCGAACGCAGCCAAGGT |

| **Table S5.** The antibodies used for this study | | |
| --- | --- | --- |
| Antibodies | Source | Identifier/RRID |
| SLC25A20 Polyclonal antibody | Proteintech | 19363-1-AP/AB_10642001 |
| Anti-Perilipin 3/TIP47 antibody | Abcam | ab47638/AB_881572 |
| Arginase-1 Polyclonal antibody | Proteintech | 16001-1-AP/AB_2289842 |
| CD68 Recombinant monoclonal antibody | Proteintech | 84596-4-RR |
| CD8a Recombinant monoclonal antibody | Proteintech | 85977-4-RR |
| Anti-Ly6g antibody | Abcam | ab238132/AB_2923218 |
| CEACAM8/CD66b Antibody | Novus | NB100-77808/AB_2077866 |
| Anti-ZDHHC18 antibody | Abclonal | A15199/AB_2762091 |
| FITC anti-mouse CD45 antibody | Biolegend | Clone no. 30-F11/AB_312972 |
| PerCP/Cyanine5.5 anti-mouse CD3 antibody | Biolegend | Clone no. 17A2/AB_1595597 |
| Alexa Fluor® 700 anti-mouse CD4 antibody | Biolegend | Clone no. GK1.5/AB_493698 |
| PE/Cyanine7 anti-mouse CD8a antibody | Biolegend | Clone no. 53-6.7/AB_312760 |
| APC anti-mouse CD25 antibody | Biolegend | Clone no. PC61/AB_312860 |
| APC/Fire™ 750 anti-mouse CD19 antibody | Biolegend | Clone no. 6D5/AB_2572119 |
| Brilliant Violet 421™ anti-mouse F4/80 antibody | Biolegend | Clone no. BM8/AB_10901171 |
| Brilliant Violet 510™ anti-mouse/human CD11b antibody | Biolegend | Clone no. M1/70/AB_2561390 |
| PE anti-mouse Ly-6G antibody | Biolegend | Clone no. 1A8/AB_1186104 |
| APC anti-mouse LAG-3 antibody | Biolegend | Clone no. C9B7W/AB_10639935 |
| PE/Dazzle™ 594 anti-mouse PD-1 antibody | Biolegend | Clone no. 29F.1A12/AB_2566005 |
| Brilliant Violet 421™ anti-mouse CTLA-4 antibody | Biolegend | Clone no. UC10-4B9/AB_10901170 |
| APC anti-mouse Ki-67 antibody | Biolegend | Clone no. 16A8/AB_2561929 |
| PE/Cyanine7 anti-mouse Arginase 1 antibody | Invitrogen | Clone no. A1exF5/AB_2734841 |
| CD11b Monoclonal antibody | Proteintech | 66519-1-Ig/AB_2881882 |
| Anti-FLAG tag monoclonal antibody | Sigma-  Aldrich | F1804, Clone M2/AB_262044 |
| MPO Polyclonal antibody | Proteintech | 22225-1-AP/AB_2884058 |
